# Synthesis and Investigation of Physicochemical and Microbial Properties of Composites Containing Encapsulated Propolis and Sea Buckthorn Oil in Pectin Matrix

**DOI:** 10.3390/ijms26178664

**Published:** 2025-09-05

**Authors:** Liliana Woszczak, Gohar Khachatryan, Karen Khachatryan, Mariusz Witczak, Anna Lenart-Boroń, Klaudia Stankiewicz, Kinga Dworak, Greta Adamczyk, Agata Pawłowska, Ireneusz Kapusta, Marcel Krzan, Monika Godlewska, Magdalena Krystyjan

**Affiliations:** 1 Laboratory of Nanotechnology and Nanomaterials, Faculty of Food Technology, University of Agriculture in Krakow, Al. Mickiewicza 21, 31-120 Krakow, Poland; liliana.woszczak@urk.edu.pl (L.W.); karen.khachatryan@urk.edu.pl (K.K.); 2 Department of Food Analysis and Quality Assessment, Faculty of Food Technology, University of Agriculture in Krakow, Al. Mickiewicza 21, 31-120 Krakow, Poland; gohar.khachatryan@urk.edu.pl; 3 Department of Food Industry Engineering and Instrumentation, Faculty of Food Technology, University of Agriculture in Krakow, Al. Mickiewicza 21, 31-120 Krakow, Poland; mariusz.witczak@urk.edu.pl; 4 Department of Microbiology and Biomonitoring, University of Agriculture in Krakow, Mickiewicza Ave. 24/28, 30-059 Krakow, Poland; anna.lenart-boron@urk.edu.pl (A.L.-B.); klaudia.kulik@student.urk.edu.pl (K.S.); 5 Medical Microbiological Laboratory, Diagnostyka S.A., Na Skarpie 66, 31-913 Krakow, Poland; kingawyrzykowska12@gmail.com; 6 Department of Food Technology and Human Nutrition, Institute of Food Technology and Nutrition, University of Rzeszow, 4 Zelwerowicza St., 35-601 Rzeszow, Poland; gadamczyk@ur.edu.pl (G.A.); agpawlowska@ur.edu.pl (A.P.); ikapusta@ur.edu (I.K.); 7 Jerzy Haber Institute of Catalysis and Surface Chemistry, Polish Academy of Sciences, 30-239 Krakow, Poland; marcel.krzan@ikifp.edu.pl; 8 Institute of Human Nutrition Sciences, Warsaw University of Life Sciences, Nowoursynowska 159c Street 32, 02-776 Warsaw, Poland; monika_hanula@sggw.edu.pl; 9 Department of Carbohydrates Technology and Cereal Processing, Faculty of Food Technology, University of Agriculture in Krakow, Al. Mickiewicza 21, 31-120 Krakow, Poland

**Keywords:** nanocapsules, propolis, sea buckthorn oil, pectin, biocomposites

## Abstract

This study explored the synthesis and characterization of pectin-based composites containing encapsulated propolis and sea buckthorn oil. Both propolis and sea buckthorn oil are well known for their antioxidant and antimicrobial properties. To mitigate their sensitivity to environmental degradation, these compounds were encapsulated within a pectin matrix. The composites were prepared using an emulsification technique and subsequently for their physicochemical properties via scanning electron microscopy (SEM), ultraviolet–visible spectroscopy (UV-Vis), Fourier-transform infrared spectroscopy (FTIR), and differential scanning calorimetry (DSC), as well as color and mechanical testing. The results showed that freeze-dried samples exhibited heterogeneous, bubble-like structures containing nanocapsules (800–2000 nm), whereas for the film samples, the capsules were visibly embedded within the matrix. The study shows that this three-component system exhibits synergistic potential. Encapsulation significantly improved the UV barrier properties and the antioxidant activity of the nanocomposites, which demonstrated greater antioxidant capacity. Microbiological assays revealed that the pectin-based composites containing encapsulated propolis and sea buckthorn oil exhibited strong antibacterial activity, particularly against Gram-positive bacteria such as *Streptococcus* and *Staphylococcus* spp. The composites also demonstrated hydrophobic surface characteristics and reduced crystallinity, which correlates with their potential for controlled release. These results underscore the applicability of pectin–propolis–sea buckthorn oil composites as effective natural preservatives or functional ingredients in food systems, due to their high antioxidant and antimicrobial efficacy.

## 1. Introduction

The rapid development of food technology has led to the increasing use of nanotechnology in the food sector [1,2], particularly in the design of functional foods and active packaging systems. This is due to the possibility of using nanostructured food ingredients (affecting the quality of food products) and food nanosensors (allowing for the verification of the quality of food products) [3,4]. One of the key directions in functional food development is enrichment and increasing the amount of bioactive and health-promoting components in traditional foods. Bioactive compounds present in food products not only enhance the nutritional value of the products but also, by means of their antioxidant properties, contribute to extending the shelf life of foodstuffs.

Through food, micro- and macro-elements are supplied; they are essential for the proper functioning of the human body. Moreover, the presence of potential substances in food products that act preventively against diseases has become a new trend in food products. Natural bioactive compounds, thanks to a number of therapeutic properties, especially high antioxidant potential [5,6], have found their application in the prevention and/or treatment of metabolic [7,8] or cardiovascular diseases [9,10,11], urinary tract infections [12,13], degenerative diseases [14,15], or in the control of several forms of cancer [16,17,18,19]. However, in order to benefit from the multifunctionality of bioactive compounds, such as extending the shelf life of food products and influencing the improvement/protection of health, it is necessary to provide protection against the external environment which can induce degradative effects on the structure of the compounds [20], which consequently lead to their degradation. Due to their chemical instability, the direct use of pure bioactive compounds in food and medicinal products is severely limited, as they often exhibit poor solubility, are prone to rapid release, low bioavailability, and rapid degradation, resulting in the loss of health-promoting properties [21].

The solution may be the use of nano- and micro-encapsulation processes, which protect bioactive compounds from the negative effects of environmental conditions, especially oxidative stress [22]. This approach not only enhances the stability of sensitive compounds [23] but can also improve their health-promoting and therapeutic effects [24], while simultaneously masking undesirable flavors and/or odors [25]. Encapsulation (both micro and nano) of biologically active compounds allows for the development of new products with better physicochemical properties, further enriching the product with new bioactive properties [26,27]. Various encapsulation techniques, including complexation, freeze-drying, spray-drying, extrusion, and supercritical anti-solvent drying and coacervation, can be employed to obtain nano/microcapsules. These capsules consist of a solid or liquid core surrounded by a polymer coating, which isolates the active compounds from the environment. In addition, nanocapsule shells provide a natural barrier against agents/compounds that can damage the substance inside the nanocapsules [23,28]. By selecting the appropriate type of shell, it is possible to modify the level of solubility of the capsules, the rate of release, and to determine the site of release, as well as the length of action [22,29]. The current trend toward combining the use of natural ingredients with environmental protection is contributing to the search for or modification of the use of currently known natural compounds in nanotechnology-based sectors of various industries.

By selecting the appropriate type of coating, it is possible to modify the degree and rate of capsule solubility and to determine the site where the capsule should release its valuable contents [22,28,29]. The choice of matrix that will constitute suitable wall material is extremely difficult and important, particularly in view of its subsequent application. The latest trends, focusing on ecology and waste reuse on the one hand, and on the exclusive use of substances of natural origin that would simultaneously meet the requirements for capsule wall material on the other, limit the possibility of using every polymer. A material that constitutes a significant production by-product is fruit peels, e.g., from apples or citrus fruits, which are primarily a source of pectin [30]. Pectins are classified as biodegradable, renewable, and environmentally friendly biomacromolecules [31]. They belong to water-soluble heteropolysaccharides, consisting of β-(1-4)-d-galacturonic acid linked to galactose and rhamnose [32]. Due to their gelling, thickening and stabilizing properties, pectins are widely used as ingredients in a number of food products (particularly as structuring agents or components of edible films) [33]. The development of a matrix incorporating pectin would not only contribute to environmental protection but would also enable the use of capsules in novel food and/or pharmaceutical matrices.

Recently, there has been growing interest in the use of bee products such as propolis, beeswax, and royal jelly. Among these, propolis has been extensively studied as a natural substance for food enrichment and health applications. It is a resinous material collected by worker bees from the terminal buds and exudates of plants, which is then mixed with beeswax and bee salivary secretions to form a viscous, aromatic solid [34]. Thanks to large-scale clinical research, propolis has become widely used in pharmaceutical and nutraceutical formulations [35]. Due to the potential health-promoting action of functional foods containing bioactive compounds, substances/extracts of natural origin characterized by a high content of polyphenolic compounds are being sought. Propolis, produced from bee glue and used in folk medicine, represents an interesting component for capsule formulation. It is known for having a range of crucial and effective antibacterial, anti-viral, and antifungal actions, particularly observed in respiratory disorders (it inhibits the development of microorganisms such as *Staphylococcus aureus*; *S. caprae*; *Streptococcus pyogenes*; group B streptococci; *Enterobacter* spp.; *Escherichia coli*; *Pantoea* sp.; and *Acinetobacter radioresistens*). Furthermore, conducted studies have demonstrated anti-inflammatory, hepatoprotective, antioxidant, anti-hemorrhagic, anti-parasitic, anti-tumor, and anti-edematous properties; it stimulates epithelial regeneration, reduces cholesterol, revitalizes, detoxifies, and tones [36,37]. The composition of bee products is determined by the geographical location of the bee colony, botanical sources, and bee species, as well as the location and time of harvest. The overall composition of propolis is about 50% resins, 30% from wax, 10% from essential oils, 5% pollen, and 5% from other substances and materials, including organic compounds [38]. Propolis is about 850 compounds, including phenols (flavonoids and phenolic acids and their esters), terpenes, alcohols, aromatic aldehydes, fatty acids, stilbenes, steroids, and lignans [39].

Propolis has medicinal properties, which is why it is in high demand as a dietary supplement due to the properties it possesses, as well an ingredient in functional foods and is minimally processed [40,41,42].

To ensure a comprehensive capsule composition, besides bioactive compounds—essential for the proper functioning of the body—polyunsaturated fatty acids and vitamins are necessary. Sea buckthorn oil, an extract of sea buckthorn seeds and flesh, is a potential functional food product [43]. It occurs as a thick, dark brown liquid with a characteristic odor and taste. It contains unsaturated fatty acids, carotenoids, plant sterols, and vitamins A, K, and E [44]. The content of unsaturated fatty acids in sea buckthorn oil ranges from 62.5 to 67.0%, mainly comprising oleic acid, linoleic acid, and linolenic acid [45,46]. It has antioxidant, anti-inflammatory, anti-atherosclerotic, hypoglycemic, and immune-enhancing properties [47]. In addition, it has been shown to possess antibacterial properties, particularly against Gram-positive bacteria, and contributes to the prevention of infections and the promotion of wound healing. These effects are attributed to its unique composition of bioactive lipids and polyphenols, which can disrupt microbial membranes and modulate inflammatory responses [43]. However, due to its characteristic scent and sensitivity to light and heat leading to oxidative processes, the use of sea buckthorn oil has limitations. The degradation of vegetable oils can be prevented by using encapsulation, thus protecting various types of nutrients from adverse changes [48,49].

The development of capsules exclusively comprising substances of natural origin would enable the following: (a) A reduction in food waste through the utilization of fruit peels (pectins); (b) the incorporation of bioactive compounds influencing the improvement of bodily functions by leveraging the documented health-promoting properties of propolis and sea-buckthorn oil; and (c) the extension of food product shelf-life facilitated by the numerous antioxidant compounds present in the capsule components. Accordingly, the aim of the work was to develop such capsules, with a focus on their physicochemical, antioxidant, and antimicrobial properties. Their antibacterial activity against upper respiratory tract bacterial isolates was evaluated to assess their potential as natural functional ingredients for food preservation and respiratory health support. Additionally, the potential application of the developed capsules as natural food additives, offering an alternative to synthetic preservatives, was assessed.

## 2. Results

### 2.1. SEM

**Figure 1:**
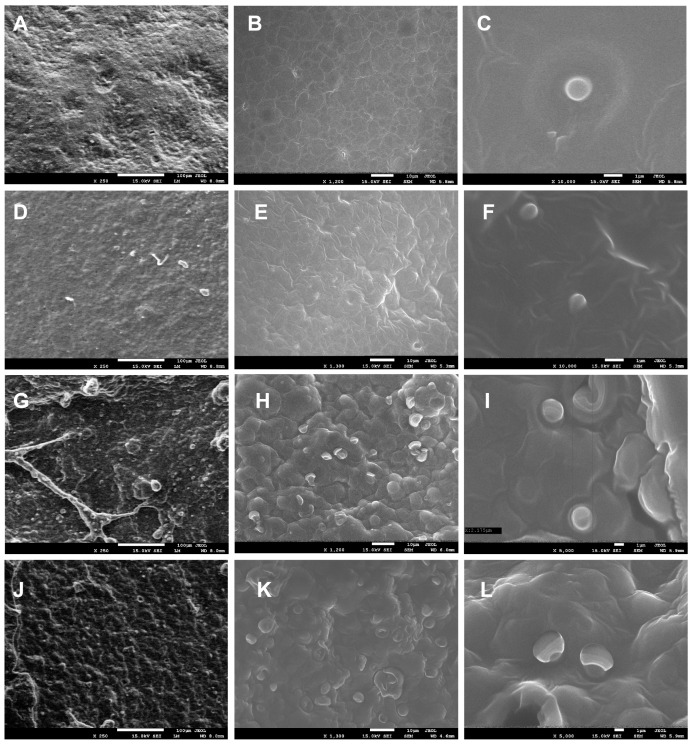
SEM images of P1 (**A**–**C**) and P2 (**D**–**F**) film samples at magnifications of 250×, 1200×, 10,000×, 250×, 1300×, and 10,000×, respectively, and for freeze-dried samples P1 (**G**–**I**) and P2 (**J**–**L**) at magnifications of 250×, 1200×, 5000×, 250×, 1300×, and 5000×, respectively.

Scanning electron microscopy (SEM) was performed on P1 and P2 samples (biocomposites containing encapsulated propolis and sea buckthorn oil in a pectin matrix, with P2 containing a higher concentration of propolis and sea buckthorn oil than P1; the detailed preparation procedure is described in Section 3.2.1), both in film and freeze-dried forms, to investigate surface morphology and characterize the resulting nano- and microstructures. Figure 1 presents representative micrographs captured at three magnification levels.

For the P1 and P2 film samples at 250× magnification, the surface appeared relatively homogeneous and slightly undulating (Figure 1A,D), contrasting with the freeze-dried samples, which exhibited a heterogeneous, bubble-like structure (Figure 1G,J). At 1200–1300× magnification, spherical structures/capsules were uniformly distributed across the surface of the freeze-dried samples (Figure 1H,K), while at 5000× magnification, capsules with a diameter of approximately 2000 nm were clearly observed for P1 (Figure 1I), with slightly larger ones for P2 (Figure 1L). In film samples, capsules were not visible on the surface at 1200× magnification (unlike in freeze-dried samples) (Figure 1B,E), as they were embedded within the polysaccharide matrix. However, at 10,000× magnification, spherical capsules measuring 800–1000 nm in size were revealed (Figure 1C,F). It was observed that the concentration of nanoemulsions has a demonstrable impact on the dimensions of the resulting capsules. Capsules in sample P1 exhibited a mean diameter approximately twice that of the capsules in sample P2.

### 2.2. UV-Vis Spectroscopy

**Figure 2:**
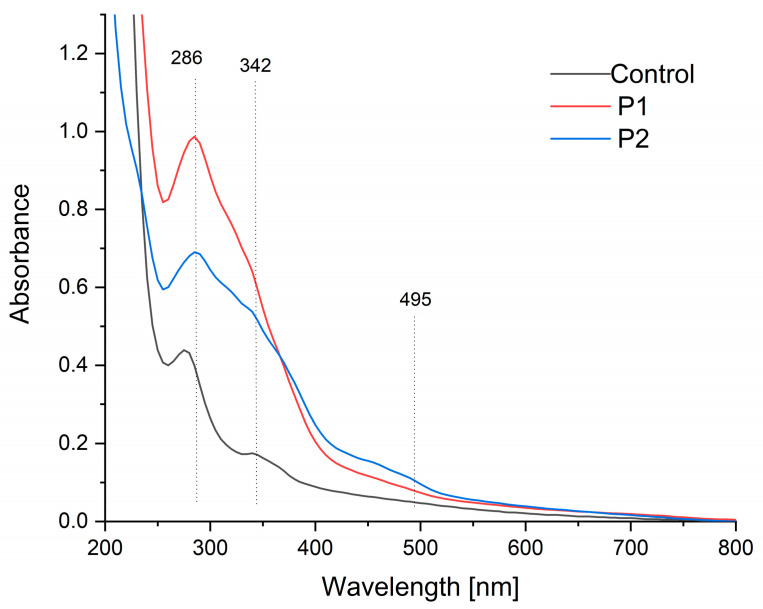
UV-Vis spectra of the pectin film (control sample) and pectin composites containing an emulsion of propolis and sea buckthorn oil (P1 and P2).

Figure 2 presents the UV-Vis absorption spectra of the fabricated films. The control sample displayed a primary absorbance peak within the 275–290 nm range, alongside secondary peaks at 320 nm and 370 nm (Figure 2). These peaks are characteristic of pectin and correspond to organic compounds such as polyphenols, amino acids (tryptophan, tyrosine, and phenylalanine, centered near 280 nm), and flavonoids (approximately 325 nm), indicating the presence of proteins and phenolic compounds in the pectin structure [50,51].

In contrast, samples encapsulated with propolis and sea buckthorn oil exhibited enhanced absorption across the 250–550 nm range compared to the control. Notably, the 280 nm peak intensity increased substantially, with a slight redshift to 286 nm observed in samples P1 and P2. Additionally, a distinct new absorption maximum emerged within the 450–500 nm range. These changes are consistent with the incorporation of propolis and sea buckthorn oil, as their constituent compounds (e.g., flavonoids, carotenoids, and oils) contribute to broader UV absorption [52,53,54,55].

A quantitative UV barrier assessment was performed by calculating the integrated area under the UV-Vis spectra (280–400 nm). The measured areas were found to be 30 a.u. for the control sample (K), 90 a.u. for P1, and 72 a.u. for P2. The findings indicate that encapsulation led to a 67% (P1) and 58% (P2) reduction in UV transmission compared to the control, substantiating a substantial enhancement of the barrier properties.

### 2.3. FTIR

**Figure 3:**
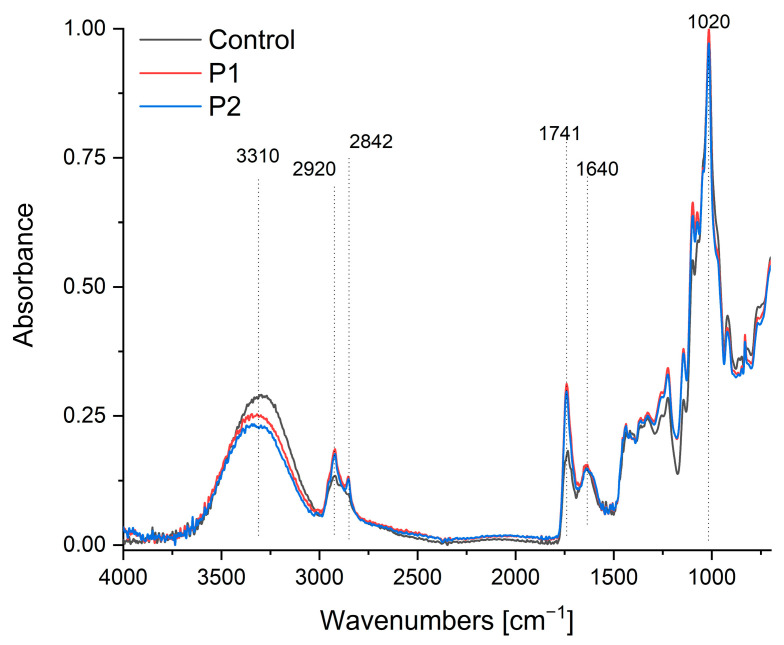
FTIR-ATR spectra of the pectin film (control sample) and pectin composites containing an emulsion of propolis and sea buckthorn oil (P1 and P2).

Figure 3 presents the FTIR-ATR spectra (750–4000 cm^−1^) of pectin films and composites encapsulating propolis–sea buckthorn oil emulsions. The control spectrum exhibits characteristic pectin bands: the broad absorption at 3310 cm^−1^ corresponds to pyranose ring vibrations and O-H stretching [56], though this undergoes a significant hypsochromic shift to ~3280 cm^−1^ in P1/P2 composites with reduced intensity, indicating enhanced hydrogen bonding between pectin’s hydroxyl groups and the polyphenolic compounds in propolis. Concurrently, the peak at 2930 cm^−1^, attributed to sp^3^ C-H stretching vibrations in the control, shows a hypsochromic displacement to 2915 cm^−1^ in the composites, suggesting constrained methylene group mobility due to hydrophobic interactions with terpenoid constituents in propolis. The broad carbonyl region (1639–1745 cm^−1^) reveals critical interactions: while the control’s band at 1640 cm^−1^ signifies free carboxyl groups in pectin [57,58], P1/P2 composites exhibit both a new prominent peak at 1741 cm^−1^ (νC=O of sea buckthorn triglycerides [55,59]) and a bathochromic shift in the carboxyl signal to 1635 cm^−1^, with an approximately 40% intensity reduction. This demonstrates successful lipid incorporation alongside carboxylate protonation, likely through ionic interactions with flavonoid moieties in propolis. Furthermore, the enhanced absorption at 1452 cm^−1^—associated with C-H deformation and the aromatic ring vibrations of propolis flavonoids [28,60]—displays an 8 cm^−1^ bathochromic shift compared to pure propolis references, signifying π-π stacking interactions between pectin’s pyranose rings and the flavonoid aromatic systems. The characteristic pyranose ring vibration at 1020 cm^−1^ [57,58] shows a 12% intensity reduction and peak broadening (FWHM increase from 18→24 cm^−1^) in the composites, evidencing glycosidic bond distortion due to the hydrophobic encapsulation of bioactive compounds within the restructured pectin matrix. Notably, new peaks at 2920 cm^−1^ and 2842 cm^−1^ (asymmetric and symmetric CH_2_ stretching from sea buckthorn oil [55,59]) confirm lipid integration, though their attenuated intensity relative to pure oil spectra suggests partial shielding by the pectin–propolis network. Collectively, these spectral modifications—particularly the quantified peak displacements in the hydroxyl (Δν = −30 cm^−1^), carbonyl (Δν = −5 cm^−1^), and aromatic (Δν = +8 cm^−1^) regions—demonstrate multi-mechanistic interactions: hydrogen bonding mediates pectin–propolis integration, hydrophobic forces stabilize lipid components, and π-orbital stacking enhances flavonoid retention. This molecular-level compatibility explains the composite’s enhanced functional properties, including improved thermal stability and bioactive protection.

### 2.4. Thickness and Mechanical Properties

The biocomposites exhibited significant differences in thickness, ranging from 0.362 to 1.404 mm, despite the same volume of solution being poured into the Petri dishes. This variation is due to the enrichment of the samples with solids [61,62], which remained in the composite after drying (Table 1).

The observed trend was proportional to the amount of emulsion incorporated into the pectin gel. Some studies confirm that the presence of oil in the film composition can reduce water evaporation, which may affect not only its thickness but also its mechanical properties [63]. The incorporation of emulsion into the pectin gels weakened the structure of the biocomposites, resulting in a statistically significant decrease in tensile strength. The P1 and P2 biocomposites exhibited a significantly higher stiffness and, consequently, lower elongation compared to the control sample made from pectin alone. The mechanical properties of biocomposites are related to the molecular structure of the matrix. The incorporation of the lipid phase into the polymer matrix causes structural discontinuities in its structure, which explains the reduced extensibility and a lower resistance to the break of the biocomposites [64,65]. Tarique et al. confirm that the incorporation of plasticizers into the polymer matrix significantly influences the tensile strength of biocomposite films [66]. Vegetable oils and other ingredients can also act as plasticizers, similar to conventional agents such as glycerol or sorbitol. The extent and effectiveness of their plasticizing action depend on their concentration and the presence of other components in the formulation [67]. According to de Araújo et al. [68], the plasticizing effect may improve elongation at the break; however, the magnitude and direction of this effect depend on the concentration of the plasticizer used. An excessive amount may lead to the opposite outcome. Osuna and coworkers [69] noticed that the addition of honey to pectin gel caused a strong plasticizing effect on the obtained films, which contributed to the decrease in TS. According to the authors, this effect could be due to honey sugars, which reduce the number of hydrogen bonds and reduce friction between pectin chains. Marangoni Junior et al. [70] also observed a weakening of the pectin matrix structure as a result of adding green propolis extract.

Sea buckthorn oil exerted a notable influence on the mechanical properties of the biocomposites, with higher concentrations leading to a progressive decrease in both tensile strength and material stiffness. The increase in the elasticity of the P2 biocomposite relative to P1 confirms the effect of oil additions on elongation at the break (EAB) parameter. However, we are unable to compare the obtained results with others, as there are no literature data available on such a combination of ingredients in the form of a nano- or microemulsion. Numerous studies describe the effects of propolis, honey, and other bee products on the pectin matrix [69,70,71], yet none consider the impact of adding sea buckthorn oil to these components.

The additives introduced into the pectin matrix had a significant impact on the mechanical properties of the composites; however, no synergistic enhancement of the pectin structure was observed. Reduced TS and EAB values suggest increased susceptibility of the biocomposite to comminution, such as during milling, which may represent a significant advantage for its application in food technology.

### 2.5. Surface Color

The color parameters are presented in Table 2. The L* parameter is used to describe the brightness (luminance) of a color [72]. The control sample showed high luminance (93.19); therefore, it reflects a lot of light, and the color perceived by the observer is bright. In contrast, the samples containing sea buckthorn oil and propolis (P1 and P2) showed a darkening of color (56.39 and 49.79, respectively). The values recorded were fewer than those obtained by Osuna and coworkers [69]; however, they found a similar trend. The increasing concentrations of honey and propolis ethanolic extract in the pectin gels decreased the luminosity (L*) of the films. Moreover, the effect of propolis was more pronounced than that of honey, and the values decreased from 85.40 to 83.47.

The presence of an oil emulsion in the polysaccharide matrix affects its darkening, because the fat droplets scattering light reduce the transparency of the matrix. The observed effect will depend on the size of the particles and their concentration [73]; hence, the observed differences in P1 and P2 biocomposites. The brightness of the biocomposites is also affected by the presence of substances rich in pigments that occur in propolis and sea buckthorn oil. Further results confirm that the biocomposites are a rich source of phenolic compounds. Sample P2 contains up to 40% more phenolics than sample P1 (Table 8). Thickness is also an important factor influencing the perceived brightness of an object. In the analyzed biocomposites, significant differences in this parameter were observed. The P2 biocomposite exhibited more than twice the thickness of the P1 sample (Table 1), which affected the degree of incident light reflection. A similar relationship has also been reported by other researchers [74,75].

Analysis of the remaining color parameters revealed that the dominant color of the P1 composite was orange-yellow, as indicated by the positive values of the a and b coordinates [76]. The P2 biocomposite exhibits a more orange hue but lower color saturation compared to P1, which may result from differences in composition. Sample P1 is distinguished by having the most saturated color (C > 56). The dark orange color of the biocomposites results from the presence of various natural pigments and phenolic compounds found in propolis. Among the most common are chalcones (np. pinocembrin) from the flavonoid group, as well as phenolic acids such as ferulic, caffeic, and coumaric acid [77].

The presence of these compounds was also confirmed in the P1 and P2 biocomposites (Table 8). The intense yellow-orange color of sea buckthorn fruits is attributed to a variety of pigments. In the analyzed composites, phenolic acids, flavonoids, and flavonols were identified as the main color-contributing compounds (Table 8), which is further supported by data reported in the literature [78].

### 2.6. Analysis DSC

**Figure 4:**
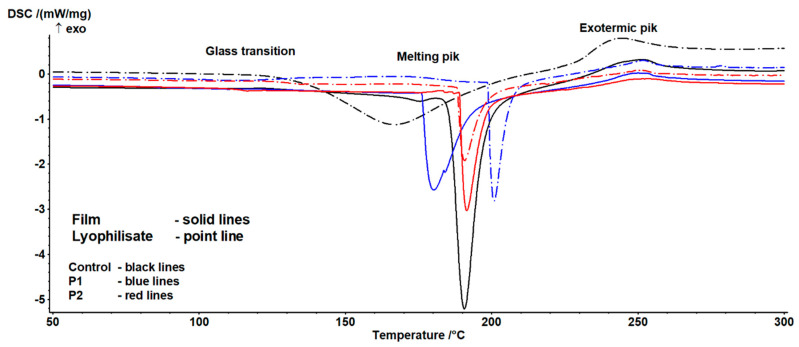
Example thermograms of the examined samples.

The results of the DSC analysis of the investigated samples are presented in Table 3 and Table 4. Example thermograms are shown in Figure 4.

The occurrence of three characteristic transitions was identified. The characteristics of the first indicate the presence of a glass transition phenomenon (Table 3). This transition exhibited a complex nature, arising on one hand from the sample history and relaxation phenomena, and on the other from the potential overlap of the glass transition of the main components in the studied formulations. Significant differences were also observed between lyophilisates and composites. In the former case, the transition process was less complex than in the latter, indicating a substantial influence of the drying technology on the value of this parameter. Significant variability depending on the sample was also noted. Increasing the proportion of the emulsion altered the nature of the transition and enhanced susceptibility to the drying technique, though this effect depended on the sample form (lyophilisate vs. composite). For lyophilisates, a continuous increase in transition heat and characteristic temperatures was observed, whereas for films, after an initial rise, a decline in the analyzed parameters was noted. This suggests significant interactions between the emulsion content and drying technique. It is important to highlight that the glass transition temperature is strongly dependent on the water content and availability. According to Iijima [79], native pectin is a crystalline polymer, but when a crystalline sample is melted, a stable amorphous form is generated. The authors observed a glass transition occurring at 37 °C, with this value influenced by the degree of esterification (DE) and moisture content. Conversely, heating propolis yields a relatively complex thermogram, attributed to the presence of multiple crystalline components that melt during heating. In this case, peaks were identified between 53 °C and 153 °C [80,81].

The second transition is associated with the melting of crystalline structures (Table 4). Here, a statistically significant influence of composition and form on temperature and enthalpy values was observed. Notably, enthalpy decreased with increasing emulsion content, suggesting a reduction in the proportion of crystalline structures. Temperature trends were less straightforward: while the end temperature of the transition showed no significant variation, the onset and peak temperatures exhibited a minimum for composites and a continuous increase for lyophilisates. This may result from changes in the pectin gel-to-emulsion ratio, which, combined with differing drying techniques, variably affects crystalline structure formation.

According to Huang et al. [82], pure pectin films display inferior thermal stability due to the plasticizing effects from hydration processes. Incorporating biopolymers can modify the thermal properties of pectin-based films. The addition of propolis reduced melting enthalpy, a similar effect to that reported by Nisar et al. [83], who developed an active pectin-based film with clove essential oil (CEO). The inclusion of CEO significantly lowered melting enthalpy, which the authors attributed to its positive impact on thermal stability by reducing the heat generated during pectin degradation [83]. The final transition of an exothermic nature showed no significant variability. It may thus be linked to pectin degradation, the content of which remained consistent across samples. This process occurs between 200 °C and 280 °C and depends on molecular parameters, the degree of modification, and physical state [84,85].

### 2.7. Zeta Potential and Particle/Aggregate Sizes

The addition of the emulsion containing propolis and sea buckthorn oil resulted in a substantial increase in particle size, from approximately 100 nm in the control sample to 4440 nm (P1) and 6250 nm (P2) (Table 5), indicating spontaneous aggregation and the formation of larger microcapsular structures. This size increase can be attributed to the higher content of the lipid phase and the encapsulation process within the pectin matrix, which promotes the formation of stable, multilayered assemblies. Concurrently, a decrease in the zeta potential was observed (from −18 mV to −14.5 mV and −12.5 mV), likely due to the presence of non-ionic components in the emulsion and the shielding effects of the polymer coating. Despite the moderate surface charge, steric stabilization provided by the biopolymer seems to play a crucial role in preventing aggregation in dry and semi-solid formulations.

The zeta potential values measured for our pectin-based nanocomposites ranged approximately between −15 and −22 mV. According to Hunter [86], zeta potential magnitudes below ±30 mV suggest that electrostatic repulsion alone may not suffice to prevent particle aggregation, potentially compromising colloidal stability over time. Delgado et al. [87]. further emphasize that such values indicate a limited electrical double layer repulsion, and that additional stabilizing mechanisms—such as steric effects—are likely to dominate.

Although the measured values suggest only moderate electrostatic stabilization, the observed colloidal stability may be enhanced by steric and hydration layer effects provided by the biopolymer matrix. Pectin, as a polysaccharide with a high molecular weight and hydrophilic character, can form extended polymeric shells around particles, reducing attractive interactions through steric hindrance. Such stabilization is particularly important in freeze-dried or semi-solid formulations, where particle mobility is already suppressed. As discussed by both Hunter and Delgado, zeta potential alone is not a sufficient indicator of stability, especially in complex or polymer-rich matrices.

From a shelf-life perspective, the relatively low zeta potential may pose limitations in aqueous dispersions, especially under variable environmental conditions. Therefore, future work will explore stabilization strategies such as pH adjustment, ionic strength control, or the incorporation of charged biopolymers or polyelectrolyte layers to enhance the dispersion stability and extend the shelf life of food and pharmaceutical applications.

In addition to surface charge, particle size is a critical parameter influencing encapsulation performance. SEM and DLS analyses showed that the capsule diameters ranged from ~800 nm to ~2000 nm, with a narrow polydispersity index (PDI < 0.3), indicating good particle uniformity. This size range is consistent with that reported by Gouin [88] as optimal for the encapsulation of bioactive compounds, providing a balance between retention efficiency, protection of sensitive cargo, and controlled release behavior.

Narrow particle size distribution contributes to uniform encapsulation, reduces variability in diffusion properties, and supports stable barrier characteristics against oxygen or light. Although not directly assessed in this study, such uniformity can also enhance mechanical and functional integration in composite matrices, as observed in related encapsulation systems. The observed differences between composite types (e.g., P1 vs. P2) are likely attributable to variations in emulsification conditions and biopolymer content, which affect droplet formation and particle growth. Smaller capsules may offer a faster release due to larger surface-area-to-volume ratios, while larger structures may prolong the release period and enhance barrier function.

In conclusion, the combination of the moderate zeta potential, steric stabilization by the pectin matrix, and narrow, well-defined particle size distribution supports both the colloidal and functional stability of the composites. High encapsulation efficiency and morphological uniformity further indicate that the system is suitable for active delivery applications. We acknowledge, however, that the lack of pH and conductivity data in this study limits the full electrokinetic interpretation of the results. These measurements will be included in future studies to strengthen dispersion characterization and reproducibility.

### 2.8. Wettability and Free Surface Energy

All samples, both the control sample and the emulsion-modified test material, show hydrophobic properties (water contact angle at about 80°) (Table 6). Surface free energy testing with two liquids, ionic and non-ionic, confirms this conclusion, showing virtually zero polar interactions in the tested samples (Table 6). The samples differ slightly from the control material and among themselves, with the most non-hydrophobic being sample one (P1), to which only 30 g of emulsion was added, and sample P2, where 60 g of emulsion was added to the starting (control) material for intermediate properties, between P1 and the control sample.

### 2.9. Antioxidant Properties

#### 2.9.1. Antioxidant Properties and Total Phenolic Content

The values of parameters illustrating the antioxidant potential of the analyzed samples are presented in Table 7.

Significant differences in antioxidant potential were observed among the control, P1, and P2 samples, as assessed by ABTS^+^, DPPH, and FRAP assays (*p* < 0.05). The control exhibited low antioxidant activity, with values of 0.77, 0.08 and 0.21 mmol TE/100 g, respectively, for the ABTS^+^, DPPH, and FRAP methods. The incorporation of propolis and sea buckthorn oil into the pectin gel markedly enhanced antioxidant capacity. In the ABTS^+^ assay, P2 reached 108.90 mmolTE/100 g, approximately 1.5-fold higher than P1 (67.93 mmolTE/100 g). The differences between P2 and P1 were even more pronounced in the DPPH (~1.7-fold) and FRAP (~1.8-fold) assays. The observed trend was consistent with the total phenolic content (TPC), which was lowest in the control (35.47 mg GAE/100 g) and strongly correlated with antioxidant activity across all methods.

The total phenolic content (TPC) in the P1 biocomposites (37,726.26 mg GAE/100 g) was approximately 100-fold higher than in the control sample, whereas the P2 sample exhibited a value around 200-fold higher (7077.59 mgGAE/100 g) (Table 1). These results indicate that the TPC was strongly influenced by the concentration of enrichment substances incorporated into the pectin matrices.

#### 2.9.2. Evaluation of Phenolic Compounds by UPLC-Q-TOF-MS

To determine the phytochemical profile of the polyphenols of methanol extracts of the preparations, UPLC-Q-TOF-MS analyses were performed using the ‘‘on-line” method. The retention times t_R_, [M − H]^−^, MS/MS fragments, and UV λ_max_ of the identified components are shown in Table 8.

Thirty-eight phenolic compounds were identified, mainly belonging to acids and esters, of which 47.4% of the identified compounds had a concentration above 1000 µg/g of composite (caftaric acid, pinobanksin-5-methyl ether, apigenin, kaempferol, pinobanksin, galangin-5-methyl ether, coumaric acid deriv., caffeic acid benzyl ester, pinocembrin, galangin, pinobanksin-3-o-acetate, caffeic acid phenylethyl ester, methoxy-chrysin, caffeic acid cinnamyl ester, pinobanksin 3-o-propionate, caffeic acid deriv., and acetylated coumaric acid deriv.). The highest concentration of phenolic compounds in a composite was identified for caffeic acid benzyl ester (10,599.40–14,340.08 µg/g); pinocembrin (6581.06–6446.82 µg/g); galangin (8039.77–13,908.89 µg/g); and pinobanksin-3-O-acetate (5039.57–7607.64 µg/g), for P1 and P2, respectively.

The source of protocatechuic acid, ellagic acid, and salicylic acid derivatives is probably sea buckthorn oil [89]. On the other hand, phenolic compounds (pinobanksin-7-methyl ether-5-O-p-hydroxyphenylpropionate, galangin-5-methyl ether, and apigenin) may originate from the propolis fraction [77]. Phenolic compounds such as ferulic acid, caffeic acid, or coumaric acid derivatives are present in both propolis and sea buckthorn oil. However, the predominance of markers specific to propolis—such as pinobanksin esters, isorhamnetin, pinocembrin, and methoxy-chrysin—indicates that propolis is the main source of phenols in the developed composites [90,91,92,93,94,95]. However, an analysis of the phenolic compound profile conducted by Medana et al. [96], Osés et al. [97], and Duca et al. [98] also demonstrated the presence of other phenolic compounds (sakuranetin, rutin, or naringenin) in propolis, which were not identified in the developed composites. However, the literature data confirm that the observed differences in the phenolic profile result from the region in which the research material was obtained. Regional diversity and abiotic and biotic conditions will influence the variability of biological material [89,99].

### 2.10. Microbiology

The growing threat of antibiotic resistance, exacerbated by the overuse of antibiotics, particularly during global health crises such as the COVID-19 pandemic, has intensified the need to search for innovative solutions not only to fight but, even more importantly, to prevent bacterial infections [100]. In this context, we evaluated the antibacterial properties of pectin-based composites containing encapsulated propolis and sea buckthorn oil, targeting bacterial strains isolated from the human upper respiratory tract. Propolis has long been recognized for its anti-viral, anti-inflammatory, antibacterial, antifungal, antioxidant, and antiseptic properties. This is due to the individual and synergistic action of propolis compounds, such as galangin, flavonoids, and pinocembrin [101,102]. These compounds act synergistically to disrupt bacterial membranes, inhibit biofilm formation, and interfere with microbial enzyme systems. A number of multifaceted antibacterial mechanisms of propolis have been reported, including the disruption of membrane potential, inhibition of ATP production, impairment of RNA and DNA synthesis, and interference with bacterial mobility and biofilm formation [103]. Sea buckthorn, although less extensively studied, exhibits antibacterial and antioxidant properties [104,105,106]. The chemical composition of sea buckthorn fruits and seeds can vary depending on, e.g., the plant variety, cultivation methods, climatic conditions etc., but the major active compounds listed as having anti-inflammatory, antibacterial, and/or antioxidant properties include: vitamin C, polyphenols, and flavonoids [105]. These components can destabilize microbial membranes and modulate inflammatory responses. Zielińska & Nowak [89] reported that the unique fatty acid composition of sea buckthorn oil—including palmitoleic acid and gamma-linolenic acid—contributes to its ability to penetrate biological membranes, promote skin regeneration, and protect against infections and inflammation by modulating prostaglandin synthesis.

**Figure 5:**
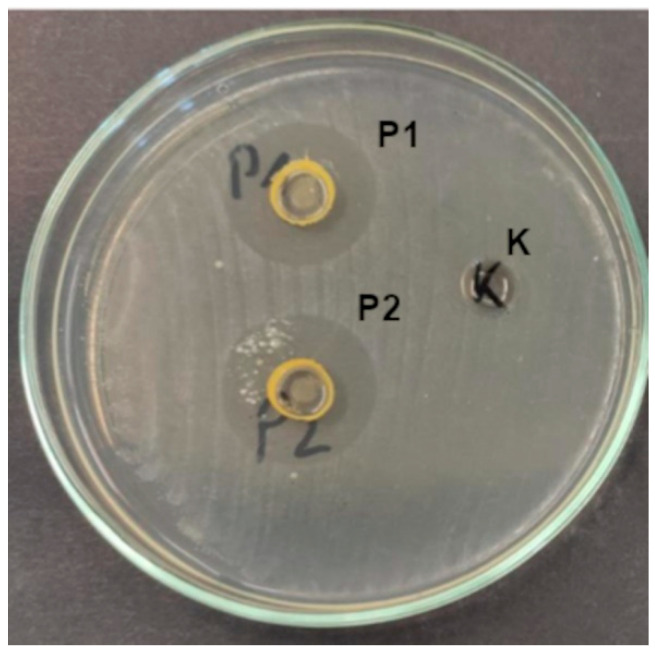
Growth inhibition zones of *S. pyogenes* under the influence of composites (K—control, P1, and P2).

In our study, 35 bacterial isolates representing 10 taxa, isolated from the human upper respiratory tract, were used in the analyses of antibacterial activity of the prepared composites in two concentrations (smaller P1 and higher P2). The P1 composite inhibited the growth of 30 isolates (constituting 85.7%), while the P2 composite inhibited the growth of 31 isolates (i.e., 88.6%) (Supplementary Table S1, Figure 5), with mean growth inhibition zones of 12.8 mm and 14.29 mm, respectively (Supplementary Table S1).

The observed stronger antibacterial activity of P2 compared to P1 can be attributed to the higher concentration of the active emulsion (propolis and sea buckthorn oil) in the P2 formulation. This aligns with the well-established principle that the antimicrobial efficacy of natural extracts often increases with concentration, up to the point of saturation. The differences observed in our study (i.e., greater overall mean growth inhibition zones, as well as greater growth inhibition zones in individual tax and a higher number of inhibited isolates) suggest the dose-dependent enhancement of antibacterial action. The increased activity of P2 may also reflect a synergistic interaction between the bioactive compounds in propolis and sea buckthorn oil. At higher concentrations, these compounds—such as flavonoids, phenolic acids, and unsaturated fatty acids—may more effectively disrupt bacterial membranes, inhibit enzyme systems, and interfere with quorum sensing and biofilm formation. This is particularly relevant for Gram-positive bacteria, which were more susceptible in both formulations. Our findings are consistent with previous studies showing that increasing the concentration of propolis or sea buckthorn extracts enhances antimicrobial activity. For example, Przybyłek and Karpiński [102] reported a concentration-dependent inhibition of *E. coli* and *S. aureus* by ethanolic propolis extracts. Similarly, Sandulachi et al. [105] demonstrated that higher doses of sea buckthorn pulp extract more effectively inhibited both Gram-positive and Gram-negative bacteria. Although the difference between P1 and P2 was not statistically significant, both formulations showed significantly greater activity than the control (*p* < 0.05), confirming their antimicrobial efficacy. This trend suggests that further increasing the emulsion content or optimizing the encapsulation matrix could yield even more potent antimicrobial effects.

**Figure 6:**
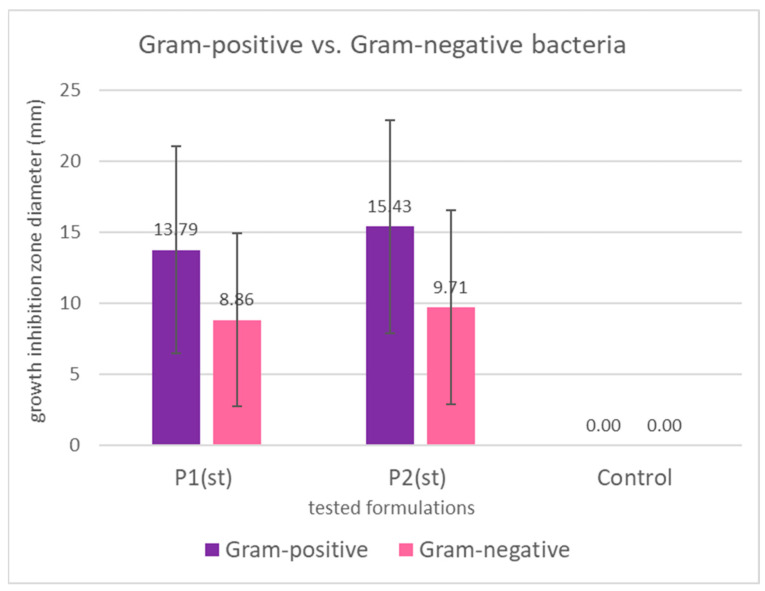
Mean growth inhibition zones in mm (bars represent standard deviations) for Gram-positive and Gram-negative bacteria caused by the application of propolis/sea buckthorn composites. The differences are not statistically significant both for P1 (*p* = 0.109) and for P2 (*p* = 0.076).

Interestingly, Gram-positive bacteria were more susceptible than Gram-negative ones (Figure 6), consistent with previous findings [102,105,107]. This is likely due to structural differences in the bacterial cell wall. Gram-negative bacteria possess an outer membrane rich in lipopolysaccharides and phospholipids, which limits the penetration of hydrophobic compounds such as those found in propolis and sea buckthorn oil [105]. Also, Gram-negative species may produce hydrolytic enzymes that degrade active compounds, further reducing bacterial susceptibility [102].

**Figure 7:**
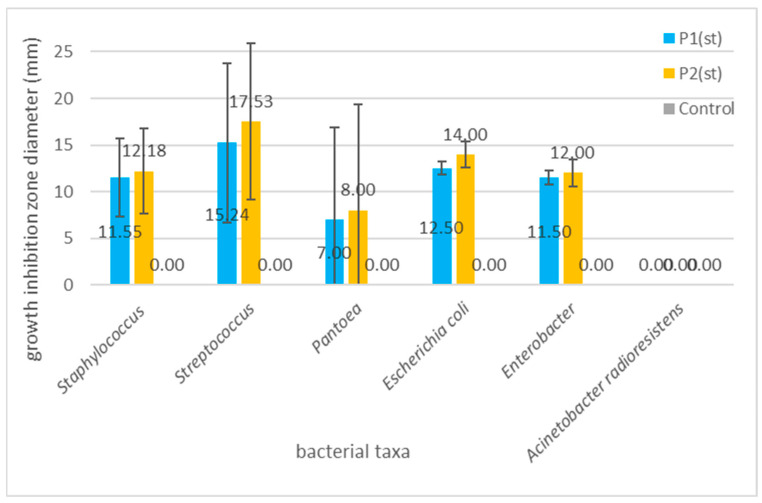
Graph showing the differences in the mean growth inhibition zones (bars represent standard deviations) for the examined bacterial taxa. The differences are statistically not significant (*p* = 0.245 for P1; *p* = 0.097 for P2), with the exception of the differences between *Streptococcus* spp. and *A. radioresistens* (LSD test: *p* = 0.044 for P1; 0.023 for P2).

Figure 7 clearly points to the highest susceptibility of *Streptococcus* spp., followed by *Staphylococus* spp.; both are key pathogens in tonsillitis, pharyngitis, and other types of upper respiratory tract inflammation [108]. *Streptococcus* spp., e.g., *S. mutans*, and *S. gordonii* have been widely reported as associated with dental caries, periodontal disease, or oral ulcers [28]. Our findings align with those of Tanuğur Samanci et al. [109], who reported a strong activity of Anatolian propolis against *Streptococcus pyogenes* and *Staphylococcus aureus*, suggesting its potential in otorhinolaryngological applications. Importantly, even though group A streptococci are well-recognized common causes of pharyngitis and tonsillitis, it has been recently pointed out that although acute tonsilitis is typically caused by a single microbial species, recurrent tonsillitis may be a consequence of polymicrobial infection [110]. Cavalcanti et al. [111]. suggested that antibiotic resistant *Staphylococcus aureus* that colonizes even non-infected tonsils can be responsible for failure in tonsillitis therapy; thus, these two bacterial taxa, i.e., *Streptococcus* and *Staphylococcus aureus*, should be treated as the most important targets of novel antimicrobial agents’ development. The third most reactive genus, *Pantoea*, has recently been reported as a recurrent sinonasal pathogen [112]. The observed growth inhibition caused by the applied composites suggests that they can be used as a promising novel food additive or medical product for prophylaxis of the upper respiratory tract, as well as oral cavity infections and conditions.

Antimicrobial and antioxidant properties of propolis, as well as sea buckthorn and their compounds, are important not only in terms of their positive impact on human health but for the food industry in terms of the shelf life extension of foods with propolis and/or sea buckthorn additives [106,113]. Przybyłek and Karpiński [102] presented a high activity of propolis against foodborne pathogens, such as *Escherichia coli* and *Enterobacter*. In our study, *Escherichia coli* and *Enterobacter* also showed a promising reaction to the application of the composites in both concentrations (mean growth inhibition of *E. coli* caused by P2 was 14 mm, while that of *Enterobacter* was 12 mm). As for sea buckthorn, Sandulachi et al. [105]. demonstrated that fruit pulp, extracts, and powder inhibited the growth of both Gram-positive (*Bacillus subtilis*, *S. aureus*) and Gram-negative (*Salmonella Typhimurium*, *E. coli*) bacteria that are among important foodborne pathogens. Overall, the antimicrobial activity observed in our composites supports their dual potential: as functional ingredients for respiratory health and as natural food additives capable of replacing synthetic preservatives. With regard to the increasing interest of consumers in using foods without or with the minimum addition of synthetic additives, the antibacterial properties observed in our experiments provide evidence that the obtained product can satisfy the needs of food producers and consumers. However, what needs to be mentioned here is that further studies are needed to explore the stability of these effects in complex food systems and under gastrointestinal conditions.

## 3. Materials and Methods

### 3.1. Materials

Material include the following: amidated apple pectin 100% (Batom.pl Józef Leśniak, Krakow, Poland); propolis (purified and concentrated ethanolic extract of propolis—P, 90.12% d.m., stored in a dark bottle, in refrigeration conditions (4 °C)) obtained from Laboratorium Bio-Farmaceutyczne (Krakow, Poland); sea buckthorn oil (Sanbios Sp. z o.o., Gliwice, Poland); glycerol used as a plasticizer, purchased from F.H.U. DOR-CHEM (Cracow, Poland); and ethyl alcohol 96%, p.a. grade.

### 3.2. Methods

#### 3.2.1. Preparation of Composites

***I. Preparation of pectin gel:*** 40.0 g of pectin was gradually added to 960.0 g of deionized water. The mixture was heated at 70 °C for 2 h with vigorous stirring (700 rpm; Heidolph RZR 2020, Heidolph Instruments GmbH & Co. KG, Germany). Subsequently, the sample was stirred at 23 °C for 24 h. Finally, 10.0 g of glycerol was incorporated and stirred for another 30 min.

***II. Preparation of emulsion:*** 20.0 g of propolis was dissolved in 30.0 g of ethyl alcohol. Then 50.0 g of sea buckthorn oil was added, and the mixture was homogenized using an ultrasonic processor (15 min, 20 kHz; Sonopuls HD 4200, Bandelin, Germany).

***III. Preparation of Control sample:*** To 350.0 g of pectin gel, 60.0 g of water was added and mixed. The gel was then divided into three portions. The initial portion was subjected to freeze-drying, while the subsequent portion was dispensed into 120 × 120 mm square Petri dishes and dried at 37 °C for 24 h in a forced-circulation oven UN110 (Memmert, Schwabach, Germany) for physicochemical analysis. The third portion was rehydrated with water (to a total mass of 136.7 g), gelled, and reserved for microbiological testing.

***IV. Preparation of P1 nanocomposite:*** 30.0 g of water was added to 350.0 g of pectin gel and mixed. Subsequently, 30.0 g of emulsion (from Step II) was gradually added to the solution, followed by homogenization (10 min, 12,000 rpm, Polytron PT 2500E, Kinematica AG, Malters, Switzerland). The product underwent the following processing steps. The initial portion was subjected to freeze-drying, while the subsequent portion was cast into 120 × 120 mm Petri dishes and air-dried at room temperature (23 °C) for a duration of 24 h to facilitate physicochemical analysis. The third portion was then rehydrated (to a mass of 136.7 g), gelled, and reserved for subsequent microbiological testing.

**Figure 8:**
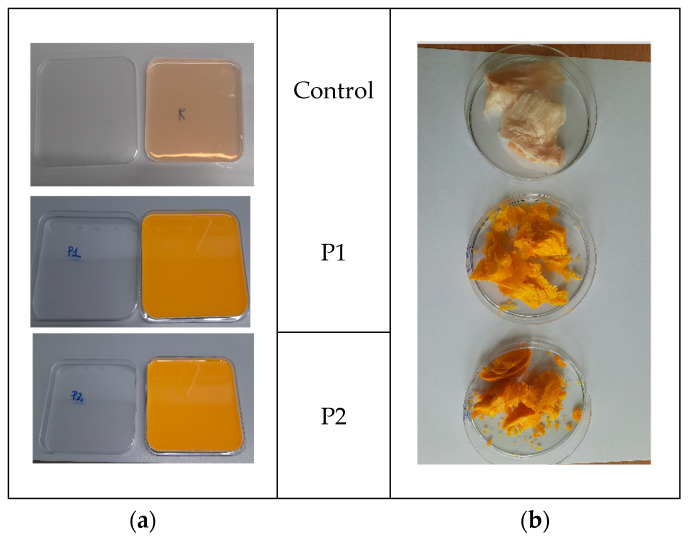
Images of the study material (**a**) in a hydrogel pectin matrix and (**b**) a lyophilized form.

***V. Preparation of P2 nanocomposite:*** 60.0 g of emulsion (obtained from Step II) was gradually added to 350.0 g of pectin gel, followed by homogenization (10 min, 12,000 rpm, Polytron PT 2500E, Kinematica AG, Malters, Switzerland). The material was processed identically to P1 (freeze-drying, air-drying, and rehydration protocols). The images of what the gels and lyophilized forms of the preparations look like are shown in Figure 8.

#### 3.2.2. SEM Miscroscopy

The size and morphology of the resulting nano/microcapsules were analyzed using a JEOL 7550 scanning electron microscope (Akishima, Tokyo, Japan), equipped with a secondary electron detector (SE). The imaging process was conducted at an accelerating voltage of 15 kV, employing a spot size of 1.0 nm. Prior to the collection of measurements, the samples were sputtered (K575X Turbo Sputter Coater, Quorum Technologies, Laughton, England, UK) with 20 nm of chromium (Cr) to enhance the conductivity of the samples.

#### 3.2.3. UV-Vis Spectroscopy

The spectra were recorded using a scanning spectrophotometer (SHIMADZU TCC-260, Kyoto, Japan) in the range of 200–700 nm. The obtained films (8 × 40 mm^2^ strips) were measured in a quartz cuvette (10 mL, 10 mm thick quartz cells). An empty cuvette was used as a reference sample.

#### 3.2.4. FTIR Spectroscopy

FTIR spectra of the obtained films were measured using a Mattson 3000 FT-IR spectrophotometer equipped with a ReFractance 30SPEC 30-angle reflectance overlay and MIRacle ATR from PIKE Technologies Inc. (Madison, WI, USA). Measurements were made at 4 cm resolution in the infrared region of 4000–750 cm^−1^. FTIR spectra have undergone comprehensive processing, including baseline correction (automatic polynomial fitting), ATR absorption correction, and vector normalization. The aforementioned procedures were performed using Omnic 9 software (v9.12.1002, Thermo Fisher Scientific, Waltham, MA, USA).

#### 3.2.5. Thickness Measurement

The thickness of composites was measured with a micrometer, catalog no. 805.1301 (Sylvac SA, Crissier, Switzerland), with a 0.001 mm resolution [114]. The sample thickness was the average of five measurements performed in various places within the gauge length area.

#### 3.2.6. Mechanical Properties of Composites

Dry composites were conditioned in desiccators at 25 °C and 52% relative humidity (RH) by using saturated solutions of magnesium nitrate-6-hydrate for 48 h prior to analysis. The samples for textural analysis were prepared according to ISO standards [115] and determined using the TA-XT plus texture analyzer (Stable Micro Systems, Haslemere, UK). Films were cut into 35 × 6 mm^2^ strips and put into holders. The initial grip separation between holders was 20 mm, and the rate of grip separation was 2 mm/min. Tensile strength (TS) was calculated by dividing tensile force (maximum force at rupture of the film) by the cross-section area of the film. The percentage of elongation at the break (EAB) was calculated by dividing the elongation at rupture by the initial gauge length and multiplying by 100 [62]. The reported results were the average values of 10 replications.

#### 3.2.7. Color Measurements

The measurement of color was carried out with the use of Konica MINOLTA CM-3500d equipment (Konica Minolta Inc., Tokyo, Japan), with a 30 mm diameter window, using a reference D65 illuminant/10° observer. The results were expressed using the CIELab system. The following parameters were determined: L* (L* = 0 black, L* = 100 white), a*—share of the green color (a* < 0) or red (a* > 0), and b*—share of blue (b* < 0) or yellow (b* > 0). The measurements were taken on a standard white background [62]. The experiment was repeated five times.

In addition, the following C* and h* parameters were calculated. Chroma (C*) or saturation describes the degree of difference in hue compared to a gray color with the same luminosity. The higher the saturation values, the higher the color intensity of the samples perceived by humans. This parameter can be calculated using the following formula [116]:

$$C^{*}=\sqrt{{a^{*}}^{2}+{b^{*}}^{2}}$$

Hue angle (h*) is the degree value that corresponds to the three-dimensional color diagram (i.e., 0 for red, 90 for yellow, 180 for green, and 270 for blue) as seen by the human eye [117] and can be calculated by the following equation:

$$h^{*}={tan}^{-1}\left(\frac{b^{*}}{a^{*}}\right)$$

#### 3.2.8. Thermal Analysis

Approximately 5 mg of the sample was weighed and sealed into aluminum pans. Subsequently, the samples were heated from 25 °C to 400 °C at a rate of 10 °C/min. The empty aluminum pan was used as a reference. The tests were performed with the DSC 204F1 Phoenix differential scanning calorimeter (Netzsch, Germany). The parameters of the observed thermal transition were calculated with Proteus Analysis software (v. 8.1.2, Netzsch, Germany). The analyses were performed, at last, in two replications.

#### 3.2.9. Dynamic Light Scattering (DLS) Measurements of Zeta Potential and Particle/Aggregate Sizes

The nanocomposite was dissolved in distilled water. After dissolution and mixing on a magnetic stirrer, the samples were placed in cells allowing for the simultaneous measurement of nanoparticle sizes and zeta potentials (DTS 1070). Zeta potential was calculated from the electrophoretic mobility of particles using the Smoluchowski model. The results are expressed as an average from three consecutive measurements with 20 runs.

The particle/aggregate sizes were measured using the Malvern Zetasizer Nano ZS apparatus with disposable measurement cells (DTS 0012, Malvern, UK). The measurement was carried out in a state-of-the-art Zetasizer Nano-ZS Malvern instrument with 10 runs.

#### 3.2.10. Wettability and Free Surface Energy Determination

In our study, we used the Drop Shape Analyzer Kruss DSA100M optical contact angle measuring instrument (KRÜSS, Hamburg, Germany, GmbH) for the evaluation of contact angles. The detailed methodology of experiments, as well as the surface free energy analysis, were presented in our previous paper [118]. We used the Owens–Wendt method [119], where two research liquids are used, ionic water and non-ionic diiodomethane. Together with the literature on the subject, it is generally accepted as the best method for polymer evaluation. An exact and detailed introduction to the Owens–Wendt methods was presented by Rudawska and co-workers [120]. All the measurements were performed in a special environmental cell at constant temperature conditions (22 °C ± 0.3) and controlled humidity. For each foil sample, at least three successive series of measurements with water and diiodomethane tests were carried out.

#### 3.2.11. Antioxidant Properties

##### Analysis of Antioxidant Properties and Total Phenolic Content

Firstly, the methanol extracts were prepared for analysis of the total phenolic content and properties of the samples. The extraction process was carried out using an ultrasonic bath (antioxidant Polsonic, Warsaw, Poland) (30 min at 25 °C). The samples were treated with 96% methanol. The obtained supernatants (methanol extracts) were used for further analysis. According to Re et al. [121], the antioxidant activity was carried out using the ABTS^+^ cation radical. The reaction mixture consisted of adding the sample (0.03 mL) and the ABTS radical solution to the water (3.0 mL). The absorbance at 734 nm, against distilled water, was measured after 6 min of reaction.

The scavenging activity was measured according to the elimination of DPPH (1,1-diphenyl-2-picrylhydrazyl) free radicals [122]. The reaction mixture consisted of adding the sample (0.5 mL) and the DPPH radical solution in methanol (2.0 mL). The absorbance at 517 nm against methanol was measured after 10 min of reaction. The analysis of antioxidant capacity using the FRAP method was performed based on Benzi and Strain [123]. Therefore, 3 mL of FRAP reagent solution was added to the analyzed samples in an amount of 0.5 mL and mixed. The absorbance of the solutions was measured after 10 min at a wavelength of 593 nm against distilled water.

Antioxidant activity determined by the ABTS^+^, DPPH, and FRAP methods was expressed in mmol TE/100 g (Trolox Equivalent—α-tocopherol analog).

The total phenolic content in the obtained samples was assessed using the Folin–Ciocalteu phenol reagent with the method described by Singleton et al. [124]. The reaction mixture contained extract (0.1 mL), Folin–Ciocalteu reagent (0.2 mL), distilled water (2.0 mL), and 20.0% (w/v) sodium carbonate anhydrous solution (1.0 mL). The absorbance of the samples was measured at 765 nm against distilled water. The total phenolic content (TPC) was expressed as gallic acid equivalents (GAEs) in milligrams per 100 g of samples.

Measurements of the ABTS, DPPH, FRAP, and TPC methods were performed in triplicate using a spectrophotometer (Nicolet Evolution 300, Thermo, Waltham, MA, USA).

##### Evaluation of Phenolic Compounds by UPLC-Q-TOF-MS

The polyphenols profile was evaluated by means UPLC-Q-TOF-MS (Waters, Milford, MA, USA). Analyses were carried out at 50 °C using a UPLC BEH C18 column (100 mm × 2.1 mm, 1.7 µm, Waters, Warsaw, Poland). The injection volume of samples was 5 µL and the isocratic flow rate was 0.35 mL/min. The mobile phase consisted of solvent A (water) and solvent B (40% acetonitrile in water, *v*/*v*). The following parameters were used for TQD: capillary voltage of 3.5 kV; con voltage of 30 V; source temperature 120 °C; desolvation temperature 350 °C; con gas flow 100 L/h; and desolvation gas flow rate of 800 L/h. Polyphenolic compounds were identified based on the following: retention time, mass-to-charge ratio, fragmentation pattern, and comparison with referenced standards and the available literature data. The quantification of polyphenolic compounds was performed by the use of the internal standard method. Analyses were performed in triplicate. All results were expressed as µg/g biocomposites.

#### 3.2.12. Microbiology

##### Isolation and Identification of Microorganisms

Swab samples were collected from the human nose, mouth, throat, and tonsils followed by inoculation on general and selective media for the isolation and preliminary identification of bacteria. Initially, the cultures were grown on Trypticase Soya Agar (incubated for 24–48 h at 36 ± 1 °C) (Biomaxima, Lublin, Poland). The resulting distinct colonies were then subcultured on the following: Baird Parker agar (Oxoid, Ceshire, UK) for the isolation and preliminary identification of coagulase-positive staphylococci, including *Staphylococcus aureus* (gray to black colonies with a clear halo after incubation for 24–48 h at 36 ± 1 °C) and on Columbia Agar with Sheep Blood Plus (Oxoid, Cheshire, UK) for the isolation and preliminary identification of streptococci (small opaque to white colonies with α or β hemolysis after incubation for 24–48 h at 36 ± 1 °C).

Subsequently, the resulting bacterial cultures were subjected to MALDI-TOF (matrix-assisted laser desorption/ionization time of flight) mass spectrometry to determine the taxonomic identity of the isolates. A total of 35 bacterial strains, belonging to 10 taxa, were selected for further analysis: *Staphylococcus aureus* (n = 10), *S. caprae* (n = 1), *Streptococcus pyogenes* (n = 11), group B streptococci (n = 6), *Enterobacter* spp. (n = 2), *Escherichia coli* (n = 2), *Pantoea* sp. (n = 2), and *Acinetobacter radioresistens* (n = 1).

##### Antimicrobial Activity of Composites

The bacteriostatic activity of the composites was evaluated using the well diffusion method. *Streptococcus* were suspended in sterile 0.85% saline solutions to obtain 1.0 MacFarland standard suspensions and then streaked onto Columbia Agar with Sheep Blood Plus (Oxoid, Cheshire, UK). For the remaining bacterial taxa, 0.5 MacFarland suspensions in 0.85% saline solutions were streaked onto Mueller–Hinton agar (Argenta, Poznań, Poland).

Wells with a 5 mm diameter were cut in the agar using a sterile cork borer, and 100 µL of UV light-sterilized (30 min) composite suspensions were poured into each well.

All cultures were incubated at 36 ± 1 °C for 24 h. After incubation, the growth inhibition zone diameters around the emulsion-filled wells were expressed in millimeters (mm).

The microbiological experiments were carried out in triplicate.

#### 3.2.13. Statistical Analysis

The statistical analysis was conducted using the Statistica software, version 13.3 (StatSoft, Tulsa, OK, USA). The one-way and two-way analyses of variance (ANOVA) and Fisher’s test were performed at *p* < 0.05 of significance.

The statistical analysis for the microbiological experiments was conducted using the Statistica software, version 13.3 (TIBCO Software Inc., PaloAlto, Santa Clara, CA, USA). The normality of the results was examined using the Shapiro–Wilk test. The distribution of results was close to normal, therefore a one-way and two-way analysis of variance with post hoc LSD test and Fisher’s test were used to assess the significance of differences between: (a) the antibacterial activity of two types of formulations and the control; (b) activity of formulations against different bacterial taxa. The significance level was set at *p* < 0.05.

## 4. Conclusions

The encapsulation of propolis and sea buckthorn oil within the pectin matrix had a negligible effect on the wettability of the matrix and slightly increased the zeta potential of the formed aggregates. However, it significantly altered the size of the aggregates. While the control sample contained nanoparticles approximately 100 nm in diameter, the samples with nanoemulsions exhibited spontaneous aggregation, leading to the formation of much larger microstructures ranging from over 4000 nm to as much as 6000 nm in size.

The biocomposites demonstrated a superior antioxidant capacity, UV protection, and antimicrobial efficacy. Increased emulsion concentration (P2) improved functional properties, suggesting dose-dependent optimization. The pectin-based composites encapsulating propolis and sea buckthorn oil exhibited broad-spectrum antibacterial activity—effectively inhibiting over 85% of human respiratory tract isolates (including *Streptococcus* spp., *Staphylococcus aureus*, and *Pantoea*) and key foodborne pathogens such as *E. coli* and *Enterobacter*—at both tested concentrations (*p* < 0.05).

The system composed of pectin, propolis, and sea buckthorn oil demonstrates synergistic potential, contributing to the improved overall performance. These findings highlight the potential of the developed composites not only as natural preventive agents against upper respiratory and oral infections but also as clean-label food additives capable of extending shelf life by replacing synthetic preservatives. The hydrophobic nature and controlled release characteristics further support their application in active food packaging or nutraceuticals. This study underscores the potential of natural, biodegradable matrices to deliver bioactive compounds, addressing challenges in food preservation and healthcare, while aligning with consumer demand for sustainable, synthetic-free products. Although the individual components used are widely recognized as safe and are commonly used in food and nutraceutical products, we acknowledge that cytotoxicity studies of the final composite formulations would be necessary to fully validate their safety for biomedical applications. Future research should therefore include in vitro and in vivo biocompatibility assessments to support potential therapeutic uses.

## Figures and Tables

**Table 1:** Mechanical properties of the biocomposites.

Sample	Thickness (mm)	TS (MPa)	EAB (%)
Control	0.362 ± 0.089 ^c^	4.32 ± 0.31 ^a^	15.90 ± 3.77 ^a^
P1	0.650 ± 0.052 ^b^	1.50 ± 0.22 ^b^	5.92 ± 0.32 ^b^
P2	1.404 ± 0.075 ^a^	0.78 ± 0.16 ^c^	8.80 ± 0.96 ^b^

The values are expressed as the mean ± standard deviation. The presence of the same superscript letter (a, b, and c) in each column indicates that there is no statistically significant difference between the values (*p* < 0.05). TS—tensile strength; EAB—percent elongation at break.

**Table 2:** Color of the biocomposites.

Sample	L* (D65)	a* (D65)	b* (D65)	C*	h*
Control	93.19 ± 1.33 ^a^	1.74 ± 0.58 ^b^	15.51 ± 3.21 ^c^	15.61 ± 3.26 ^c^	1.46 ± 0.01 ^a^
P1	56.39 ± 0.28 ^b^	25.85 ± 0.10 ^a^	50.77 ± 0.47 ^a^	56.97 ± 0.41 ^a^	1.10 ± 0.00 ^b^
P2	49.79 ± 1.01 ^c^	25.63 ± 0.58 ^a^	39.78 ± 1.83 ^b^	47.32 ± 1.83 ^b^	1.00 ± 0.01 ^c^

The values are expressed as the mean ± standard deviation. The presence of the same superscript letter (a, b, and c) in each column indicates that there is no statistically significant difference between the values (*p* < 0.05).

**Table 3:** Glass transition characteristic.

Sample	Form	T_ong_	T_midg_	T_infg_	T_endg_	−∆c_p_
		°C	°C	°C	°C	°C
Control	Composite	71.1 ± 2.8 ^ab^	81.2 ± 0.3 ^b^	75.2 ± 1.5 ^ab^	98.3 ± 9.8 ^b^	0.100 ± 0.009 ^b^
	Lyophilisate	52.5 ± 2.1 ^a^	57.2 ± 1.9 ^a^	59.6 ± 0.2 ^a^	62.7 ± 0.4 ^a^	0.053 ± 0.009 ^a^
P1	Composite	89.1 ± 23.3 ^bc^	103.5 ± 10.1 ^c^	98.3 ± 19.7 ^bc^	110.2 ± 6.2 ^bc^	0.309 ± 0.012 ^e^
	Lyophilisate	107.7 ± 17.2 ^cd^	118.1 ± 10.2 ^cd^	115.2 ± 12.5 ^cd^	121.7 ± 11.4 ^cd^	0.244 ± 0.021 ^d^
P2	Composite	95.4 ± 16.9 ^bcd^	103.2 ± 10.8 ^c^	106.1 ± 8.8 ^cd^	108.9 ± 7.4 ^bc^	0.154 ± 0.005 ^c^
	Lyophilisate	123.7 ± 0.4 ^d^	128.5 ± 0.9 ^d^	130.1 ± 2.1 ^d^	133.4 ± 2.2 ^d^	0.403 ± 0.015 ^f^
One-way ANOVA—*p*		0.014	<0.001	0.002	0.001	<0.001
Two-way ANOVA—*p*						
Factor A (Form)		0.277	0.267	0.198	0.977	0.001
Factor B (Sample)		0.005	<0.001	0.001	<0.001	<0.001
Factor A × Factor B		0.112	0.007	0.068	0.003	<0.001

Mean value of two replications ± standard deviation. Mean values signed as same letters in particular columns are non-significantly different at the 0.05 level of confidence.

**Table 4:** Melting peak parameters and exothermic transition temperatures.

Sample	Form	T_onm_	T_pm_	T_endm_	−∆H_m_	T_pe_
		°C	°C	°C	J·g^−1^	°C
Control	Composite	186.6 ± 1.0 ^bc^	191.4 ± 0.9 ^b^	198.0 ± 0.6	227.2 ± 4.7 ^d^	251.6 ± 0.1
	Lyophilisate	136.4 ± 3.5 ^a^	165.4 ± 2.0 ^a^	190.8 ± 7.6	238.5 ± 20.4 ^d^	242.2 ± 3.3
P1	Composite	175.0 ± 0.1 ^bc^	181.4 ± 0.3 ^ab^	194.1 ± 0.9	148.6 ± 14.9 ^c^	250.6 ± 0.1
	Lyophilisate	173.4 ± 14.2 ^b^	190.5 ± 12.3 ^b^	206.1 ± 6.1	104.3 ± 3.2 ^b^	249.5 ± 4.5
P2	Composite	185.7 ± 1.8 ^bc^	191.4 ± 1.7 ^b^	200.6 ± 1.8	100.0 ± 1.6 ^b^	250.5 ± 0.4
	Lyophilisate	193.5 ± 9.7 ^c^	198.3 ± 9.3 ^b^	204.6 ± 8.6	54.1 ± 13.1 ^a^	249.5 ± 1.6
One-way ANOVA—*p*		0.003	0.032	0.116	<0.001	0.092
Two-way ANOVA—*p*						
Factor A (Form)		0.019	0.455	0.369	0.004	0.045
Factor B (Sample)		0.007	0.049	0.165	<0.001	0.250
Factor A × Factor B		0.004	0.021	0.094	0.012	0.108

Mean value of two replications ± standard deviation. Mean values signed as the same letters in particular columns are non-significantly different at the 0.05 level of confidence.

**Table 5:** Particle size and zeta potential of the particles.

	Size [nm]	Zeta Potential [mV]
Control	100	−18.0
P1	4440	−14.5
P2	6250	−12.5

**Table 6:** Contact angle and surface free energy of the composites.

Sample	Contact Angle		Surface Free Energy		
	Water	Diiodomethane	Polar (mJ/m^2^)	Dispersive (mJ/m^2^)	Total Free Energy (mJ/m^2^)
Control	82.1	40.8	2.40	42.25	44.65
P1	75.5	26.8	3.55	47.23	50.78
P2	80.3	32.6	2.36	45.72	48.08

**Table 7:** Antioxidant activity and total phenolic content in pectin gel capsules and capsules from this gel enriched with propolis and sea buckthorn oil.

Samples	ABTS^+^ [mmolTE/100 g]	DPPH [mmolTE/100 g]	FRAP [mmolTE/100 g]	TPC [mgGAE/100 g]
Control	0.77 ^a^ ± 0.17	0.08 ^a^ ± 0.00	0.21 ^a^ ± 0.01	35.47 ^a^ ± 0.37
P1	67.93 ^b^ ± 3.85	12.870 ^b^ ± 1.68	13.93 ^b^ ± 0.40	3726.26 ^b^ ± 40.85
P2	108.90 ^c^ ± 3.09	22.83 ^c^ ± 3.97	25.66 ^c^ ± 4.52	7077.59 ^c^ ± 139.88

Mean values in columns denoted by different letters differ statistically significantly at *p* < 0.05.

**Table 8:** Phenolic composition of pectin-based composite samples with encapsulated propolis and sea buckthorn oil.

Compound		Rt	λ_max_	[M − H] *m*/*z*		Concentration µg/g Biocomposites	
		min	nm	MS	MS/MS	P1	P2
1	Caffeic acid	2.14	324	179	135	298.62 ± 0.00	460.69 ± 6.95
2	Protocatechuic acid	2.20	253	153	-	419.44 ± 5.61	718.78 ± 9.61
3	Coumaric acid	2.57	309	163	119	336.33 ± 10.85	414.54 ± 13.37
4	Coumaric acid	2.60	309	163	119	590.13 ± 11.92	979.91 ± 19.79
5	Ferulic acid	2.80	295, 322	193	133	217.55 ± 1.97	417.88 ± 3.77
6	Isoferulic acid	2.88	298, 320	193	133	310.09 ± 2.22	512.96 ± 3.68
8	Salicilic acid deriv.	3.28	269	331	269	284.81 ± 10.11	417.61 ± 14.82
9	Caftaric acid	3.47	321	311	149	895.90 ± 13.03	1330.31 ± 19.35
10	Elagic acid	3.52	367	301	257	323.57 ± 10.92	524.26 ± 17.70
11	Isorhamnetin/Rhamnetin	3.72	255, 355	315	300	445.25 ± 1.69	662.17 ± 2.52
12	Pinobanksin-5-methyl ether	3.80	286	285	267, 252	1808.63 ± 48.74	2719.11 ± 73.28
13	Apigenin	3.90	267, 333	269	225, 151	779.69 ± 13.83	1022.16 ± 18.13
14	Unknown	3.94	281	345	207	618.63 ± 27.89	751.75 ± 33.89
15	Kaempferol	3.97	265, 366	285	257	843.44 ± 1.23	1264.78 ± 1.85
16	Pinobanksin	4.12	291	271	253, 225	2322.89 ± 81.52	3130.48 ± 109.87
17	Kaempferol-methyl eter	4.17	267, 349	299	284	683.55 ± 6.59	977.22 ± 9.42
18	Pinocembrin-5-methyl ether	4.21	285	269	255, 227	336.45 ± 23.11	330.72 ± 22.72
19	Quercetin-dimethyl ether isomer I	4.29	255, 353	329	271	591.43 ± 6.89	686.60 ± 8.00
20	Galangin-5-methyl ether	4.51	260, 350	283	268, 239	994.51 ± 27.51	1212.05 ± 33.52
21	Isorhamnetin/Rhamnetin	4.56	255, 364	315	300	598.82 ± 7.69	736.19 ± 9.46
22	Coumaric acid deriv.	4.66	309	535	163	2727.17 ± 47.43	4043.24 ± 70.32
23	Quercetin-dimethyl ether isomer II	4.76	255, 355	329	271	522.74 ± 13.03	635.66 ± 15.84
24	Caffeic acid benzyl ester	4.98	267, 315	269	178, 134	10,599.40 ± 207.28	14,340.08 ± 280.43
25	Pinocembrin	5.08	290	255	227	6581.06 ± 275.06	6446.82 ± 269.45
26	Galangin	5.10	265, 300 sh, 360	269	241, 227	8039.77 ± 150.25	13,908.89 ± 259.93
27	Pinobanksin-3-*O*-acetate	5.16	293	313	271, 253	5039.57 ± 260.62	7607.64 ± 393.42
28	Caffeic acid phenylethyl ester	5.21	296, 327	283	179, 135	1538.70 ± 61.08	2394.20 ± 95.04
29	Methoxy-chrysin	5.33	266, 310 sh, 340 sh	283	268	1089.68 ± 16.98	1576.91 ± 24.57
30	Caffeic acid cinnamyl ester	5.50	295, 321	295	253	2577.78 ± 45.32	3539.57 ± 62.22
31	Pinobanksin 3-*O*-propionate	5.63	293	327	271, 253	2335.50 ± 88.21	3185.17 ± 120.30
32	Caffeic acid deriv.	5.72	322	501	353	918.47 ± 27.76	1273.08 ± 38.48
33	Unknown	5.81	291	319	269	423.85 ± 5.43	521.90 ± 6.69
34	Pinobanksin-7-methyl ether-5-*O*-p-hydroksyphenylpropionate	5.90	291	475	433, 415	364.01 ± 17.34	596.58 ± 28.42
35	Acetylated coumaric acid deriv.	5.97	310	325	279, 163	1647.42 ± 3.68	2272.45 ± 5.07
36	Pinobanksin-3-*O*-butyrate or isobutyrate	6.08	293	341	271, 253	1521.73 ± 30.40	2232.98 ± 44.61
37	Unknown	6.20	288	325	265	729.49 ± 41.45	1114.72 ± 63.34
38	Unknown	6.22	267	325	269	1296.64 ± 3.84	1761.43 ± 5.22
39	Unknown	6.29	266, 300 sh, 350	325	265	343.52 ± 2.41	488.61 ± 3.43
40	Pinobanksin-3-*O*-pentanoate or 2-methylbuturate	6.47	291	355	271, 253	272.16 ± 3.31	409.08 ± 4.98
41	Pinobanksin-3-*O*-hexenoate	6.54	291	367	271, 253	101.69 ± 2.20	166.00 ± 3.60
42	Pinobanksin-3-*O*-phenylpropionate hexenoate	6.59	291	403	271, 253	302.61 ± 3.19	424.45 ± 4.48
43	Pinobanksin-3-*O*-hexanoate	6.83	291	369	271, 253	143.08 ± 1.49	202.05 ± 2.20
	**Total**					65,308.53 ± 251.33	91,892.41 ± 625.46

## Data Availability

The data presented in this study are available upon request from the corresponding author.

## Supplementary Materials

The following supporting information can be downloaded at: https://www.mdpi.com/article/10.3390/ijms26178664/s1.

## References

[1] Nile S.H., Baskar V., Selvaraj D., Nile A., Xiao J., Kai G. (2020). Nanotechnologies in food science: Applications, recent trends, and future perspectives. Nano-Micro Lett..

[2] Dima C., Assadpour E., Dima S., Jafari S.M. (2020). Bioactive-loaded nanocarriers for functional foods: From designing to bioavailability. Curr. Opin. Food Sci..

[3] Jummai J.M., Balogu V.T. (2023). A Review On Novel Use Of Nanotechnology In Food And Dairy Industry To Enhanced Functional And Nutritional Qualities. http://irepo.futminna.edu.ng:8080/jspui/handle/123456789/26860.

[4] Kumar A., Samtiya M., Sharma A., Dhiman S., Krishan B., Singhi P., Dhewa T. (2024). Application of Nanotechnology to Developing Nutraceuticals and Functional Foods. Functional Foods and Nutraceuticals: Chemistry, Health Benefits and the Way Forward.

[5] Diniz do Nascimento L., Barbosa de Moraes A.A., Santana da Costa K., Pereira Galúcio J.M., Taube P.S., Leal Costa C.M., Neves Cruz J., de Aguiar Andrade E.H., Guerreiro de Faria L.J. (2020). Bioactive natural compounds and antioxidant activity of essential oils from spice plants: New findings and potential applications. Biomolecules.

[6] Zhao Y.-S., Eweys A.S., Zhang J.-Y., Zhu Y., Bai J., Darwesh O.M., Zhang H.-B., Xiao X. (2021). Fermentation affects the antioxidant activity of plant-based food material through the release and production of bioactive components. Antioxidants.

[7] Noce A., Di Lauro M., Di Daniele F., Pietroboni Zaitseva A., Marrone G., Borboni P., Di Daniele N. (2021). Natural bioactive compounds useful in clinical management of metabolic syndrome. Nutrients.

[8] Ramírez-Moreno E., Arias-Rico J., Jiménez-Sánchez R.C., Estrada-Luna D., Jiménez-Osorio A.S., Zafra-Rojas Q.Y., Ariza-Ortega J.A., Flores-Chávez O.R., Morales-Castillejos L., Sandoval-Gallegos E.M. (2022). Role of bioactive compounds in obesity: Metabolic mechanism focused on inflammation. Foods.

[9] Rangel-Huerta O.D., Pastor-Villaescusa B., Aguilera C.M., Gil A. (2015). A systematic review of the efficacy of bioactive compounds in cardiovascular disease: Phenolic compounds. Nutrients.

[10] Sharifi-Rad J., Rodrigues C.F., Sharopov F., Docea A.O., Can Karaca A., Sharifi-Rad M., Kahveci Karıncaoglu D., Gülseren G., Şenol E., Demircan E. (2020). Diet, lifestyle and cardiovascular diseases: Linking pathophysiology to cardioprotective effects of natural bioactive compounds. Int. J. Environ. Res. Public Health.

[11] Sindhu R.K., Goyal A., Algın Yapar E., Cavalu S. (2021). Bioactive compounds and nanodelivery perspectives for treatment of cardiovascular diseases. Appl. Sci..

[12] Tache A.M., Dinu L.D., Vamanu E. (2022). Novel insights on plant extracts to prevent and treat recurrent urinary tract infections. Appl. Sci..

[13] Vamanu E., Dinu L.D., Luntraru C.M., Suciu A. (2021). In vitro coliform resistance to bioactive compounds in urinary infection, assessed in a lab catheterization model. Appl. Sci..

[14] Shoaib S., Ansari M.A., Fatease A.A., Safhi A.Y., Hani U., Jahan R., Alomary M.N., Ansari M.N., Ahmed N., Wahab S. (2023). Plant-derived bioactive compounds in the management of neurodegenerative disorders: Challenges, future directions and molecular mechanisms involved in neuroprotection. Pharmaceutics.

[15] Franco G.A., Interdonato L., Cordaro M., Cuzzocrea S., Di Paola R. (2023). Bioactive compounds of the Mediterranean diet as nutritional support to fight neurodegenerative disease. Int. J. Mol. Sci..

[16] Yao Y., Zhou Y., Liu L., Xu Y., Chen Q., Wang Y., Wu S., Deng Y., Zhang J., Shao A. (2020). Nanoparticle-based drug delivery in cancer therapy and its role in overcoming drug resistance. Front. Mol. Biosci..

[17] Fonseca L.R., Silva G.R., Luís Â., Cardoso H.J., Correia S., Vaz C.V., Duarte A.P., Socorro S. (2021). Sweet cherries as anti-cancer agents: From bioactive compounds to function. Molecules.

[18] Aziz A., Noreen S., Khalid W., Mubarik F., Niazi M.K., Koraqi H., Ali A., Lima C.M.G., Alansari W.S., Eskandrani A.A. (2022). Extraction of bioactive compounds from different vegetable sprouts and their potential role in the formulation of functional foods against various disorders: A literature-based review. Molecules.

[19] Enciso-Martínez Y., Zuñiga-Martínez B.S., Ayala-Zavala J.F., Domínguez-Avila J.A., González-Aguilar G.A., Viuda-Martos M. (2024). Agro-Industrial By-Products of Plant Origin: Therapeutic Uses as well as antimicrobial and antioxidant activity. Biomolecules.

[20] ElGamal R., Song C., Rayan A.M., Liu C., Al-Rejaie S., ElMasry G. (2023). Thermal degradation of bioactive compounds during drying process of horticultural and agronomic products: A comprehensive overview. Agronomy.

[21] Ling J.K.U., Sam J.H., Jeevanandam J., Chan Y.S., Nandong J. (2022). Thermal degradation of antioxidant compounds: Effects of parameters, thermal degradation kinetics, and formulation strategies. Food Bioprocess Technol..

[22] Pateiro M., Gómez B., Munekata P.E., Barba F.J., Putnik P., Kovačević D.B., Lorenzo J.M. (2021). Nanoencapsulation of promising bioactive compounds to improve their absorption, stability, functionality and the appearance of the final food products. Molecules.

[23] Zabot G.L., Schaefer Rodrigues F., Polano Ody L., Vinícius Tres M., Herrera E., Palacin H., Córdova-Ramos J.S., Best I., Olivera-Montenegro L. (2022). Encapsulation of bioactive compounds for food and agricultural applications. Polymers.

[24] Yusuf A., Almotairy A.R.Z., Henidi H., Alshehri O.Y., Aldughaim M.S. (2023). Nanoparticles as drug delivery systems: A review of the implication of nanoparticles’ physicochemical properties on responses in biological systems. Polymers.

[25] Kurek M., Benaida-Debbache N., Elez Garofulić I., Galić K., Avallone S., Voilley A., Waché Y. (2022). Antioxidants and bioactive compounds in food: Critical review of issues and prospects. Antioxidants.

[26] Khachatryan G., Pląder J., Piechowicz K., Witczak T., Liszka-Skoczylas M., Witczak M., Gałkowska D., Duraczyńska D., Hunter W., Waradzyn A. (2024). Preparation and Study of the Physicochemical and Functional Properties of Nano/Micromicellar Structures Containing Chokeberry Fruit Pomace Extracts Using Egg White and Egg Yolk. Int. J. Mol. Sci..

[27] Turek K., Khachatryan G., Khachatryan K., Krystyjan M. (2023). An innovative method for the production of yoghurt fortified with walnut oil nanocapsules and characteristics of functional properties in relation to conventional yoghurts. Foods.

[28] Khachatryan G., Khachatryan K., Krystyjan M., Krzemińska-Fiedorowicz L., Lenart-Boroń A., Białecka A., Krupka M., Krzan M., Blaszyńska K., Hanula M. (2023). Synthesis and investigation of physicochemical and biological properties of films containing encapsulated propolis in hyaluronic matrix. Polymers.

[29] Massel V., Fang Y., Corredig M. (2021). Pectin nanoemulsions in multiple emulsions: Stability and encapsulation efficiency. Food Res. Int..

[30] Benassi L., Alessandri I., Vassalini I. (2021). Assessing green methods for pectin extraction from waste orange peels. Molecules.

[31] Jia Y., Wang C., Khalifa I., Zhu Y., Wang Z., Chen H., Liang X., Zhang H., Hu L., Yang W. (2024). Pectin: A review with recent advances in the emerging revolution and multiscale evaluation approaches of its emulsifying characteristics. Food Hydrocoll..

[32] Roy S., Priyadarshi R., Łopusiewicz Ł., Biswas D., Chandel V., Rhim J.-W. (2023). Recent progress in pectin extraction, characterization, and pectin-based films for active food packaging applications: A review. Int. J. Biol. Macromol..

[33] Chandel V., Biswas D., Roy S., Vaidya D., Verma A., Gupta A. (2022). Current advancements in pectin: Extraction, properties and multifunctional applications. Foods.

[34] Pu Y., Jiang H., Zhang Y., Cao J., Jiang W. (2023). Advances in propolis and propolis functionalized coatings and films for fruits and vegetables preservation. Food Chem..

[35] Chavda V.P., Chaudhari A.Z., Teli D., Balar P., Vora L. (2023). Propolis and Their Active Constituents for Chronic Diseases. Biomedicines.

[36] El-Sakhawy M., Salama A., Mohamed S.A.A. (2024). Propolis applications in food industries and packaging. Biomass Convers. Biorefinery.

[37] Ibáñez B., Melero A., Montoro A., San Onofre N., Soriano J.M. (2023). Radioprotective Effects from Propolis: A Review. Molecules.

[38] Adefegha S.A., Oboh G., Oluokun O.O. (2022). Food bioactives: The food image behind the curtain of health promotion and prevention against several degenerative diseases. Stud. Nat. Prod. Chem..

[39] Segueni N., Boutaghane N., Asma S.T., Tas N., Acaroz U., Arslan-Acaroz D., Shah S.R.A., Abdellatieff H.A., Akkal S., Peñalver R. (2023). Review on Propolis Applications in Food Preservation and Active Packaging. Plants.

[40] Rahman S., Talukdar N.C., Patowary K., Mohanta Y.K. (2024). Propolis from the North-East region of India exhibits potent antioxidant and anticancer activity against breast cancer cells. Biocatal. Agric. Biotechnol..

[41] Chacon W.D.C., Monteiro A.R., Verruck S., Valencia G.A. (2024). Optimization model of starch nanoparticles production loaded with phenolic compounds from green propolis extract. J. Polym. Environ..

[42] González Montiel L., León-López A., García-Ceja A., Franco-Fernández M.J., Pérez-Soto E., Cenobio-Galindo A.d.J., Campos-Montiel R.G., Aguirre-Álvarez G. (2024). Stability, content of bioactive compounds and antioxidant activity of emulsions with propolis extracts during simulated in vitro digestion. Foods.

[43] Suryakumar G., Rathor R., Singh S.N., Kumar B. (2022). Medicinal and Nutraceutical Properties of Seabuckthorn. The Seabuckthorn Genome.

[44] Samanci A.E.T., Muluk N.B., Samanci T., Cingi C. (2024). Propolis: Prevention and Healing Effects in Otorhinolaryngology.

[45] Song A., Li Y., Wang W., Hu Y., Xu J., Xu Z., Zhou L., Liu J. (2024). Revealing the effect of sea buckthorn oil, fish oil and structured lipid on intestinal microbiota, colonic short chain fatty acid composition and serum lipid profiles in vivo. Nat. Prod. Bioprospect..

[46] Wirkowska-Wojdyła M., Ostrowska-Ligęza E., Górska A., Brzezińska R., Piasecka I. (2024). Assessment of the nutritional potential and resistance to oxidation of sea buckthorn and rosehip oils. Appl. Sci..

[47] Chen Y., Cai Y., Wang K., Wang Y. (2023). Bioactive compounds in sea buckthorn and their efficacy in preventing and treating metabolic syndrome. Foods.

[48] Shen Z., Dai J., Yang X., Liu Y., Liu L., Huang Y., Wang L., Chen P., Chen X., Zhang C. (2024). Comparison of sea buckthorn fruit oil nanoemulsions stabilized by protein-polysaccharide conjugates prepared using β-glucan from various sources. Food Chem..

[49] Sun R., Zhang M., Zhao J., Lu M., Hao J., Guan X., Li C. (2024). Anti-atherosclerotic effect of sea buckthorn (*Hippophae Rhamnoides* Linn) and its molecular mechanism. J. Funct. Foods.

[50] Lin J., Meng H., Guo X., Yu S. (2022). Enhancing the emulsification and photostability properties of pectin from different sources using Genipin crosslinking technique. Foods.

[51] Lin J., Tang Z.-s., Brennan C.S., Zeng X.-A. (2022). Thermomechanically micronized sugar beet pulp: Dissociation mechanism, physicochemical characteristics, and emulsifying properties. Food Res. Int..

[52] Adaškevičiūtė V., Kaškonienė V., Kaškonas P., Barčauskaitė K., Maruška A. (2019). Comparison of physicochemical properties of bee pollen with other bee products. Biomolecules.

[53] Zhang H., Song G., Ma W., Guo M., Ling X., Yu D., Zhou W., Li L. (2023). Microencapsulation protects the biological activity of sea buckthorn seed oil. Front. Nutr..

[54] Kowalczyk D., Karaś M., Kazimierczak W., Skrzypek T., Wiater A., Bartkowiak A., Basiura-Cembala M. (2025). A Comparative Study on the Structural, Physicochemical, Release, and Antioxidant Properties of Sodium Casein and Gelatin Films Containing Sea Buckthorn Oil. Polymers.

[55] Topală C.M., Ducu C. (2014). Spectroscopic study of sea buckthorn extracts. Curr. Trends Nat. Sci..

[56] Kozioł A., Środa-Pomianek K., Górniak A., Wikiera A., Cyprych K., Malik M. (2022). Structural determination of pectins by spectroscopy methods. Coatings.

[57] Wani K.M., Uppaluri R.V. (2023). Characterization of pectin extracted from pomelo peel using pulsed ultrasound assisted extraction and acidic hot water extraction process. Appl. Food Res..

[58] Jandosov J., Alavijeh M., Sultakhan S., Baimenov A., Bernardo M., Sakipova Z., Azat S., Lyubchyk S., Zhylybayeva N., Naurzbayeva G. (2022). Activated carbon/pectin composite enterosorbent for human protection from intoxication with xenobiotics Pb (II) and sodium diclofenac. Molecules.

[59] Kala S., Sogan N., Naik S., Agarwal A., Kumar J. (2020). Impregnation of pectin-cedarwood essential oil nanocapsules onto mini cotton bag improves larvicidal performances. Sci. Rep..

[60] Ibrahim N., Zakaria A.J., Ismail Z., Ahmad Y., Mohd K. (2018). Application of GCMS and FTIR fingerprinting in discriminating two species of Malaysian stingless bees propolis. Int. J. Eng. Technol..

[61] Hovhannisyan A., Janik M., Woszczak L., Khachatryan G., Krystyjan M., Lenart-Boroń A., Stankiewicz K., Czernecka N., Duraczyńska D., Oszczęda Z. (2023). The Preparation of Silver and Gold Nanoparticles in Hyaluronic Acid and the Influence of Low-Pressure Plasma Treatment on Their Physicochemical and Microbiological Properties. Int. J. Mol. Sci..

[62] Krystyjan M., Khachatryan G., Grabacka M., Krzan M., Witczak M., Grzyb J., Woszczak L. (2021). Physicochemical, bacteriostatic, and biological properties of starch/chitosan polymer composites modified by graphene oxide, designed as new bionanomaterials. Polymers.

[63] Taqi A., Askar K.A., Nagy K., Mutihac L., Stamatin L. (2011). Effect of different concentrations of olive oil and oleic acid on the mechanical properties of albumen (egg white) edible films. Afr. J. Biotechnol..

[64] Sánchez-González L., Chiralt A., González-Martínez C., Cháfer M. (2011). Effect of essential oils on properties of film forming emulsions and films based on hydroxypropylmethylcellulose and chitosan. J. Food Eng..

[65] Gallo J.-A.Q., Debeaufort F., Callegarin F., Voilley A. (2000). Lipid hydrophobicity, physical state and distribution effects on the properties of emulsion-based edible films. J. Membr. Sci..

[66] Tarique J., Sapuan S., Khalina A. (2021). Effect of glycerol plasticizer loading on the physical, mechanical, thermal, and barrier properties of arrowroot (Maranta arundinacea) starch biopolymers. Sci. Rep..

[67] Mali S., Grossmann M.V.E., García M.A., Martino M.N., Zaritzky N.E. (2006). Effects of controlled storage on thermal, mechanical and barrier properties of plasticized films from different starch sources. J. Food Eng..

[68] deAraújo G.K.P., deSouza S.J., daSilva M.V., Yamashita F., Gonçalves O.H., Leimann F.V., Shirai M.A. (2015). Physical, antimicrobial and antioxidant properties of starch-based film containing ethanolic propolis extract. Int. J. Food Sci. Technol..

[69] Osuna M.B., Romero C.A., Rivas F.P., Judis M.A., Bertola N.C. (2025). Apple pectin based film with Apis mellifera Honey and/or propolis extract as sources of active compounds. Food Biophys..

[70] Marangoni Júnior L., Gonçalves S.d.Á., Silva R.G.d., Martins J.T., Vicente A.A., Alves R.M.V., Vieira R.P. (2022). Effect of green propolis extract on functional properties of active pectin-based films. Food Hydrocoll..

[71] Salimi A., Khodaiyan F., Askari G., Amraei A. (2025). Effect of propolis extract-loaded films made of apple pomace pectin and grass pea protein on the shelf life extension of black mulberry. Food Hydrocoll..

[72] Stanisławska N., Khachatryan G., Khachatryan K., Krystyjan M., Makarewicz M., Krzan M. (2023). Formation and investigation of physicochemical and microbiological properties of biocomposite films containing turmeric extract nano/microcapsules. Polymers.

[73] Gueli A.M., Bonfiglio G., Pasquale S., Troja S.O. (2017). Effect of particle size on pigments colour. Color Res. Appl..

[74] Muñoz-Gimena P.F., Aragón-Gutiérrez A., Blázquez-Blázquez E., Arrieta M.P., Rodríguez G., Peponi L., López D. (2024). Avocado Seed Starch-Based Films Reinforced with Starch Nanocrystals. Polymers.

[75] Ruiz-López J., Espinar C., Lucena C., de la Cruz Cardona J., Pulgar R., Pérez M.M. (2023). Effect of thickness on color and translucency of a multi-color polymer-infiltrated ceramic-network material. J. Esthet. Restor. Dent..

[76] Folentarska A., Łagiewka J., Krystyjan M., Ciesielski W. (2021). Biodegradable binary and ternary complexes from renewable raw materials. Polymers.

[77] Okińczyc P., Widelski J., Szperlik J., Żuk M., Mroczek T., Skalicka-Woźniak K., Sakipova Z., Widelska G., Kuś P.M. (2021). Impact of plant origin on eurasian propolis on phenolic profile and classical antioxidant activity. Biomolecules.

[78] Bal L.M., Meda V., Naik S., Satya S. (2011). Sea buckthorn berries: A potential source of valuable nutrients for nutraceuticals and cosmoceuticals. Food Res. Int..

[79] Iijima M., Nakamura K., Hatakeyama T., Hatakeyama H. (2000). Phase transition of pectin with sorbed water. Carbohydr. Polym..

[80] Matsuda A.H., Machado L.B., del Mastro N.L. (2002). Thermal analysis applied to irradiated propolis. Radiat. Phys. Chem..

[81] Da Silva F.C., Favaro-Trindade C.S., de Alencar S.M., Thomazini M., Balieiro J.C. (2011). Physicochemical properties, antioxidant activity and stability of spray-dried propolis. J. ApiProduct ApiMedical Sci..

[82] Huang J., Hu Z., Hu L., Li G., Yao Q., Hu Y. (2021). Pectin-based active packaging: A critical review on preparation, physical properties and novel application in food preservation. Trends Food Sci. Technol..

[83] Nisar T., Wang Z.-C., Yang X., Tian Y., Iqbal M., Guo Y. (2018). Characterization of citrus pectin films integrated with clove bud essential oil: Physical, thermal, barrier, antioxidant and antibacterial properties. Int. J. Biol. Macromol..

[84] Einhorn-Stoll U., Kunzek H. (2009). Thermoanalytical characterisation of processing-dependent structural changes and state transitions of citrus pectin. Food Hydrocoll..

[85] Filho L.B., Lima S.K.R., de S. (2024). Barbosa, H. Ultrasound-assisted extraction of pectin from Spondias purpurea L. peels residues: Optimization, characterization, and comparison with commercial pectin. J. Food Meas. Charact..

[86] Hunter R.J. (2013). Zeta Potential in Colloid Science: Principles and Applications.

[87] Delgado Á.V., González-Caballero F., Hunter R., Koopal L., Lyklema J. (2007). Measurement and interpretation of electrokinetic phenomena. J. Colloid Interface Sci..

[88] Gouin S. (2004). Microencapsulation: Industrial appraisal of existing technologies and trends. Trends Food Sci. Technol..

[89] Zielińska A., Nowak I. (2017). Abundance of active ingredients in sea-buckthorn oil. Lipids Health Dis..

[90] Falcão S.I., Vale N., Gomes P., Domingues M.R., Freire C., Cardoso S.M., Vilas-Boas M. (2013). Phenolic profiling of Portuguese propolis by LC–MS spectrometry: Uncommon propolis rich in flavonoid glycosides. Phytochem. Anal..

[91] Falcão S.I., Vilas-Boas M., Estevinho L.M., Barros C., Domingues M.R., Cardoso S.M. (2010). Phenolic characterization of Northeast Portuguese propolis: Usual and unusual compounds. Anal. Bioanal. Chem..

[92] Pellati F., Orlandini G., Pinetti D., Benvenuti S. (2011). HPLC-DAD and HPLC-ESI-MS/MS methods for metabolite profiling of propolis extracts. J. Pharm. Biomed. Anal..

[93] Saftić L., Peršurić Ž., Fornal E., Pavlešić T., Pavelić S.K. (2019). Targeted and untargeted LC-MS polyphenolic profiling and chemometric analysis of propolis from different regions of Croatia. J. Pharm. Biomed. Anal..

[94] Karagecili H., Yılmaz M.A., Ertürk A., Kiziltas H., Güven L., Alwasel S.H., Gulcin İ. (2023). Comprehensive metabolite profiling of Berdav propolis using LC-MS/MS: Determination of antioxidant, anticholinergic, antiglaucoma, and antidiabetic effects. Molecules.

[95] Nichitoi M.M., Josceanu A.M., Isopescu R.D., Isopencu G.O., Geana E.-I., Ciucure C.T., Lavric V. (2021). Polyphenolics profile effects upon the antioxidant and antimicrobial activity of propolis extracts. Sci. Rep..

[96] Medana C., Carbone F., Aigotti R., Appendino G., Baiocchi C. (2008). Selective analysis of phenolic compounds in propolis by HPLC-MS/MS. Phytochem. Anal. Int. J. Plant Chem. Biochem. Tech..

[97] Osés S.M., Marcos P., Azofra P., de Pablo A., Fernández-Muíño M.Á., Sancho M.T. (2020). Phenolic profile, antioxidant capacities and enzymatic inhibitory activities of propolis from different geographical areas: Needs for analytical harmonization. Antioxidants.

[98] Duca A., Sturza A., Moacă E.-A., Negrea M., Lalescu V.-D., Lungeanu D., Dehelean C.-A., Muntean D.-M., Alexa E. (2019). Identification of resveratrol as bioactive compound of propolis from western Romania and characterization of phenolic profile and antioxidant activity of ethanolic extracts. Molecules.

[99] Danielski R., Shahidi F. (2024). Phenolic composition and bioactivities of sea buckthorn (*Hippophae rhamnoides* L.) fruit and seeds: An unconventional source of natural antioxidants in North America. J. Sci. Food Agric..

[100] Salama S.A., Essam D., Tagyan A.I., Farghali A.A., Khalil E.M., Abdelaleim Y.F., Hozzein W.N., Mubarak M., Nasr F.A., Eweis A.A. (2024). Novel composite of nano zinc oxide and nano propolis as antibiotic for antibiotic-resistant bacteria: A promising approach. Sci. Rep..

[101] Sforcin J.M., Bankova V., Kuropatnicki A.K. (2017). Medical benefits of honeybee products. Evid.-Based Complement. Altern. Med. eCAM.

[102] Przybyłek I., Karpiński T.M. (2019). Antibacterial properties of propolis. Molecules.

[103] Bhatti N., Hajam Y.A., Mushtaq S., Kaur L., Kumar R., Rai S. (2024). A review on dynamic pharmacological potency and multifaceted biological activities of propolis. Discov. Sustain..

[104] Negi P., Chauhan A., Sadia G., Rohinishree Y., Ramteke R. (2005). Antioxidant and antibacterial activities of various seabuckthorn (*Hippophae rhamnoides* L.) seed extracts. Food Chem..

[105] Sandulachi E., Macari A., Cojocari D., Balan G., Popa S., Turculeț N., Ghendov-Moşanu A., Sturza R. (2022). Antimicrobial properties of sea buckthorn grown in the Republic of Moldova. J. Eng. Sci..

[106] Yue X.-F., Shang X., Zhang Z.-J., Zhang Y.-N. (2017). Phytochemical composition and antibacterial activity of the essential oils from different parts of sea buckthorn (*Hippophae rhamnoides* L.). J. Food Drug Anal..

[107] Rajkowska K., Rykała E., Czyżowska A. (2024). Antibacterial Effect of Sea Buckthorn (*Hippophae rhamnoides* L.) Fruit Extract on Radish Seeds Prior to Sprouting. Pol. J. Food Nutr. Sci..

[108] Ashurst J.V., Weiss E., Tristram D., Edgerley-Gibb L. (2025). Streptococcal Pharyngitis. StatPearls [Internet].

[109] Tanuğur Samanci A.E., Bayar Muluk N., Samanci T., Cingi C. (2024). Pulmonary Effects of Propolis. Propolis: Prevention and Healing Effects in Otorhinolaryngology.

[110] Jensen A., Fagö-Olsen H., Sørensen C.H., Kilian M. (2013). Molecular mapping to species level of the tonsillar crypt microbiota associated with health and recurrent tonsillitis. PLoS ONE.

[111] Cavalcanti V.P., Camargo L.A.d., Moura F.S., Melo Fernandes E.J.d., Lamaro-Cardoso J., Braga C.A.d.S.B., André M.C.P. (2019). Staphylococcus aureus in tonsils of patients with recurrent tonsillitis: Prevalence, susceptibility profile, and genotypic characterization. Braz. J. Infect. Dis..

[112] Su Y.-W., Huang W.-H., Yeh C.-F. (2022). Pantoea dispersa rhinosinusitis: Clinical aspects of a rare sinonasal pathogen. Eur. Arch. Oto-Rhino-Laryngol..

[113] Almuhayawi M.S. (2020). Propolis as a novel antibacterial agent. Saudi J. Biol. Sci..

[114] Krystyjan M., Khachatryan G., Ciesielski W., Buksa K., Sikora M. (2017). Preparation and characteristics of mechanical and functional properties of starch/Plantago psyllium seeds mucilage films. Starch-Stärke.

[115] ISO 527-1:2019. Determination of Tensile Properties–Part 1: General Principles. https://www.iso.org/standard/75824.html.

[116] Pathare P.B., Opara U.L., Al-Said F.A.-J. (2013). Colour measurement and analysis in fresh and processed foods: A review. Food Bioprocess Technol..

[117] Sant’Anna V., Gurak P.D., Marczak L.D.F., Tessaro I.C. (2013). Tracking bioactive compounds with colour changes in foods—A review. Dye. Pigment..

[118] Khachatryan G., Khachatryan K., Krystyjan M., Krzan M., Khachatryan L. (2020). Functional properties of composites containing silver nanoparticles embedded in hyaluronan and hyaluronan-lecithin matrix. Int. J. Biol. Macromol..

[119] Owens D.K., Wendt R. (1969). Estimation of the surface free energy of polymers. J. Appl. Polym. Sci..

[120] Rudawska A., Jacniacka E. (2009). Analysis for determining surface free energy uncertainty by the Owen–Wendt method. Int. J. Adhes. Adhes..

[121] Re R., Pellegrini N., Proteggente A., Pannala A., Yang M., Rice-Evans C. (1999). Antioxidant activity applying an improved ABTS radical cation decolorization assay. Free Radic. Biol. Med..

[122] Yen G.-C., Chen H.-Y. (1995). Antioxidant activity of various tea extracts in relation to their antimutagenicity. J. Agric. Food Chem..

[123] Benzie I.F., Strain J.J. (1996). The ferric reducing ability of plasma (FRAP) as a measure of “antioxidant power”: The FRAP assay. Anal. Biochem..

[124] Vl S. (1999). Analysis of total phenols and other oxidation substrates and antioxidants by means of Folin-Ciocalteu reagent. Methods Enzymol..

