# Supplementary material for: Synthesis and Investigation of Physicochemical and Microbial Properties of Composites Containing Encapsulated Propolis and Sea Buckthorn Oil in Pectin Matrix
Source: Int J Mol Sci. 2025 Sep 5;26(17):8664. doi: 10.3390/ijms26178664 (PMC12428884; doi:10.3390/ijms26178664)
Supplement: Supplementary file 1 [file ijms-26-08664-s001.zip › ijms-3684269-supplementary.pdf]

Supplementary Table S1. Growth inhibition zones of bacterial isolates, isolated from human upper respiratory tract, under the influence of composites.

	P1(st)	P2(st)	K
Staphylococcus aureus	13,00	12,00	0,00
Staphylococcus aureus	11,00	12,00	0,00
Staphylococcus aureus	15,00	17,00	0,00
Staphylococcus aureus	16,00	17,00	0,00
Staphylococcus aureus	11,00	12,00	0,00
Staphylococcus aureus	13,00	14,00	0,00
Staphylococcus aureus	11,00	12,00	0,00
Staphylococcus aureus	0,00	0,00	0,00
Staphylococcus aureus	12,00	12,00	0,00
Staphylococcus aureus	11,00	11,00	0,00
Staphylococcus caprae	14,00	15,00	0,00
Streptococcus B	15,00	18,00	0,00
Streptococcus B	27,00	29,00	0,00
Streptococcus B	13,00	17,00	0,00
Streptococcus B	11,00	12,00	0,00
Streptococcus B	0,00	0,00	0,00
Streptococcus B	12,00	12,00	0,00
Streptococcus pyogenes	15,00	17,00	0,00
Streptococcus pyogenes	35,00	40,00	0,00
Streptococcus pyogenes	0,00	14,00	0,00
Streptococcus pyogenes	13,00	16,00	0,00
Streptococcus pyogenes	14,00	15,00	0,00
Streptococcus pyogenes	13,00	18,00	0,00
Streptococcus pyogenes	13,00	14,00	0,00
Streptococcus pyogenes	23,00	24,00	0,00
Streptococcus pyogenes	20,00	15,00	0,00
Streptococcus pyogenes	14,00	15,00	0,00
Streptococcus pyogenes	21,00	22,00	0,00
Enterobacter	12,00	13,00	0,00
Enterobacter	11,00	11,00	0,00
Acinetobacter radioresistens	0,00	0,00	0,00
Escherichia coli	13,00	15,00	0,00
Escherichia coli	12,00	13,00	0,00
Pantoea	14,00	16,00	0,00
Pantoea	0,00	0,00	0,00

average	12,80	14,29	0,00
standard deviation	7,26	7,62	0,00
